# Identification of unannotated microproteins involved in endothelial cell homeostasis, dysfunction, and vascular disease

**DOI:** 10.1093/cvr/cvag110

**Published:** 2026-05-19

**Authors:** Mauro Siragusa, Johannes Graumann, Carsten Kuenne, Stefan Günther, Sylvia Jeratsch, Beyza Güven, Sebastian Süsser, Sweta Talyan, Bethlehem Bezuneh, Yves Matthess, Matteo Cartura, Xiaozhu Zhou, Haaglim Cho, Nadja Sachs, Lars Maegdefessel, Oliver J Müller, Christian Troidl, Holger M Nef, Mario Looso, Manuel Kaulich, Stefan Offermanns, Ingrid Fleming

**Affiliations:** Institute for Vascular Signalling, Centre for Molecular Medicine, Goethe University, Frankfurt am Main, Germany; Cardio-Pulmonary Institute, Frankfurt am Main and Bad Nauheim, Germany; Biomolecular Mass Spectrometry, Max Planck Institute for Heart and Lung Research, Bad Nauheim, Germany; Institute & Core Facility of Translational Proteomics, Biochemical-Pharmacological Center, Philipps-Universität Marburg, Marburg, Germany; German Center for Cardiovascular Research (DZHK), Partner Site RheinMain, Frankfurt am Main and Bad Nauheim, Germany; Bioinformatics and Deep Sequencing Platform, Max Planck Institute for Heart and Lung Research, Bad Nauheim, Germany; Bioinformatics and Deep Sequencing Platform, Max Planck Institute for Heart and Lung Research, Bad Nauheim, Germany; Biomolecular Mass Spectrometry, Max Planck Institute for Heart and Lung Research, Bad Nauheim, Germany; Institute for Vascular Signalling, Centre for Molecular Medicine, Goethe University, Frankfurt am Main, Germany; Institute of Biochemistry II, Faculty of Medicine, Goethe University, Frankfurt am Main, Germany; Bioinformatics and Deep Sequencing Platform, Max Planck Institute for Heart and Lung Research, Bad Nauheim, Germany; Institute for Vascular Signalling, Centre for Molecular Medicine, Goethe University, Frankfurt am Main, Germany; Institute of Biochemistry II, Faculty of Medicine, Goethe University, Frankfurt am Main, Germany; Institute for Vascular Signalling, Centre for Molecular Medicine, Goethe University, Frankfurt am Main, Germany; Institute for Vascular Signalling, Centre for Molecular Medicine, Goethe University, Frankfurt am Main, Germany; Department of Pharmacology, Max Planck Institute for Heart and Lung Research, Bad Nauheim; Department for Vascular and Endovascular Surgery, Technical University Munich, Klinikum rechts der Isar, Munich, Germany; German Center for Cardiovascular Research (DZHK), Partner Site Munich Heart Alliance, Munich, Germany; German Center for Cardiovascular Research (DZHK), Partner Site Munich Heart Alliance, Munich, Germany; Institute of Molecular Vascular Medicine, Technical University Munich, Munich, Germany; Department of Medicine, Karolinska Institutet and University Hospital, Stockholm, Sweden; Department of Internal Medicine V, University of Kiel, Kiel, Germany; German Center for Cardiovascular Research (DZHK), Partner Site Hamburg/Kiel/Lübeck, Kiel, Germany; German Center for Cardiovascular Research (DZHK), Partner Site RheinMain, Frankfurt am Main and Bad Nauheim, Germany; Department of Cardiology, Kerckhoff Heart & Lung Center, Bad Nauheim, Germany; German Center for Cardiovascular Research (DZHK), Partner Site RheinMain, Frankfurt am Main and Bad Nauheim, Germany; Department of Cardiology, Kerckhoff Heart & Lung Center, Bad Nauheim, Germany; Department of Cardiology & Angiology, Justus-Liebig-University Giessen, Giessen, Germany; Cardio-Pulmonary Institute, Frankfurt am Main and Bad Nauheim, Germany; Bioinformatics and Deep Sequencing Platform, Max Planck Institute for Heart and Lung Research, Bad Nauheim, Germany; Cardio-Pulmonary Institute, Frankfurt am Main and Bad Nauheim, Germany; Institute of Biochemistry II, Faculty of Medicine, Goethe University, Frankfurt am Main, Germany; Frankfurt Cancer Institute, Frankfurt am Main, Germany; Cardio-Pulmonary Institute, Frankfurt am Main and Bad Nauheim, Germany; German Center for Cardiovascular Research (DZHK), Partner Site RheinMain, Frankfurt am Main and Bad Nauheim, Germany; Department of Pharmacology, Max Planck Institute for Heart and Lung Research, Bad Nauheim; Centre for Molecular Medicine, Goethe University, Frankfurt am Main, Germany; Institute for Vascular Signalling, Centre for Molecular Medicine, Goethe University, Frankfurt am Main, Germany; Cardio-Pulmonary Institute, Frankfurt am Main and Bad Nauheim, Germany; German Center for Cardiovascular Research (DZHK), Partner Site RheinMain, Frankfurt am Main and Bad Nauheim, Germany

**Keywords:** Small open reading frames, Microproteins, RiboTag, Endothelial cell, Cardiovascular disease

## Abstract

**Aims:**

Microproteins (miPs) translated from small open reading frames (smORFs) are crucial regulators of cell function. However, the expression and function of miPs in endothelial cells and alterations in miP expression linked with inflammation and cardiovascular disease, remain largely unexplored.

**Methods and results:**

An optimized proteogenomic approach combining RiboTag RNA-sequencing and mass spectrometry of the small molecular mass proteome was utilized to identify endothelial cell-specific miPs. Heart, lung, and blood vessels from endothelial cell-specific RiboTag mice and human endothelial cells were studied under homeostatic and inflammatory conditions. We identified 2739 murine as well as 1365 intracellular and 607 extracellular human endothelial cell miPs encoded from previously non-canonical (unannotated) smORFs. Vascular inflammation induced *in vitro* by interleukin-1β (IL-1β) and *in vivo* through PCSK9 overexpression, high-fat diet, and partial carotid artery ligation significantly altered smORF expression. An additional 347 miPs were detected in human serum, 23 decreasing and 31 increasing, after cardiac damage. The expression of an inflammation-induced miP encoded by an internal smORF within the proline-serine-threonine phosphatase interacting protein 2 (PSTPIP2) transcript, that is, miP-PSTPIP2, was assessed using a custom antibody. miP-PSTPIP2 expression was upregulated in IL-1β-treated human endothelial cells, in pre-atherosclerotic murine carotid arteries and detected in carotid arteries from patients with atherosclerosis. The relevance of 250 miPs for endothelial cell growth and viability was demonstrated using a high-throughput clustered regularly interspaced Short palindromic Repeats (CRISPR)/Cas9 screen.

**Conclusion:**

Taken together, we document the existence of a large number of human and murine miPs encoded by non-canonical smORFs and their altered expression in inflammatory conditions. The identification of secreted miPs suggests that they may also exert autocrine or paracrine functions. These novel small peptides modulate cell proliferation and survival in endothelial cells and may play a significant role in human cardiovascular disease.

## Graphical Abstract

**Graphical Abstract:**
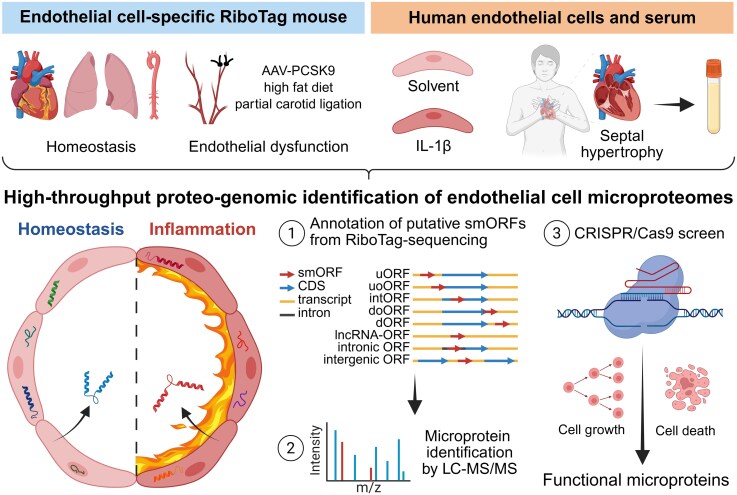

## 1. Introduction

The translation of thousands of unannotated human open reading frames (ORFs) has been extensively documented.^1–10^ The majority of these ORFs consist of 100 or fewer codons (small ORFs or smORFs) and exhibit limited evolutionary conservation.^3,11^ Seminal studies have identified ORFs based on the *in silico* three-frame translation of transcriptomes or evolutionary sequence conservation, while the majority of recent studies have applied ribosome profiling to demonstrate smORF translation.^1,2,4,12–14^ By detecting the movement of translating ribosomes along a transcript in steps of three nucleotides, that is, periodicity, ribosome profiling can be used to infer the frame of active translation.^15^ At first sight, this would seem to be the method of choice to identify smORFs, but there are important shortcomings in its application for smORF annotation. These include very high variations in data quality, depth, and sparseness, as well as the low reproducibility between different smORF detection algorithms.^16^ Also, a large number of multi-mapping reads from ribosome-protected footprints cannot be unambiguously assigned to a specific genomic location and are therefore discarded. Even though ribosome profiling has been instrumental in identifying translation events in what were thought to be ‘untranslated’ regions, that is, upstream ORFs (uORFs), downstream ORFs (dORFs), and long non-coding ORFs (lnc-RNA ORFs), it exhibits limited capacity to resolve the multiple periodicities of overlapping reading frames in protein-coding regions, which ultimately results in highly divergent smORF annotations. Indeed, a recent study elegantly documented that four different scoring algorithms applied to the same ribosome profiling dataset produced considerably divergent numbers, biotypes, and length of identified smORFs.^16^ It also reported a relatively low reproducibility across replicates. Importantly, in a separate study,^17^ many of the microproteins (miPs) translated from smORFs and whose existence was confirmed by mass spectrometry, were not picked up by ribosome profiling. Indeed, an integrated proteogenomic approach was essential to identify miPs encoded by smORFs that were either not called or had uncertain mapping by ribosome profiling.^16–18^ Another limitation that distinguishes ribosome profiling from other RNA sequencing techniques is its inability to differentiate between different cell types when ribosomes are isolated from tissues *in vivo*. Equally difficult is demonstrating that identified smORFs are translated into *bona fide* miPs. This is because the likelihood of detecting a protein in standard proteomic pipelines is proportional to its size, and smaller proteins generate fewer tryptic peptides. Although proteomic datasets deposited in repositories have in the past been successfully interrogated to identify miPs,^1–4,6,9,13,19–21^ the lack of targeted enrichment strategies for small proteins means that the actual number of miPs is likely to be markedly underestimated. Finally, the databases used to match spectra to peptides do not include miP sequences, and as a consequence, these tend to be ‘filtered out’.

Despite the challenges associated with their detection, it is clear that miPs are actively involved in biological processes ranging from cell growth to the regulation of metabolism, and are likely to play major roles in pathophysiological conditions.^10,14,21,22^ Indeed, miPs can act as signalling molecules, interact with other proteins to alter their conformation and/or activation state, as well as modulate the assembly of macromolecular complexes with other proteins or nucleic acids.^21,23,24^ In the heart, hundreds of novel miPs have been identified, and in cardiomyocytes, miPs are involved in the regulation of calcium handling,^25^ energy production by mitochondria,^22^ and contractility,^26^ as well as in heart disease.^3,27^ In contrast, the extent to which endothelial cell miPs are involved in vascular homeostasis or the initiation of vascular disease has not been explored. This is despite the previous association of vascular inflammation with the translation of 21 mitochondrial smORFs.^28^

In this study, we combined whole transcriptome RNA- and RiboTag RNA-sequencing^29^ to identify putative endothelial cell-specific smORFs in intact murine tissues *in vivo*. We focussed on highly vascularized tissues, as the endothelial cells that line blood vessels respond to signals derived from the blood and the stroma and are rapidly activated in response to acute insults. We also studied alterations in the translation of smORFs in the carotid artery endothelium in a disease-relevant model of vascular inflammation. In parallel, whole transcriptome and RiboTag RNA-sequencing studies were conducted using cultured human endothelial cells under homeostatic and inflammatory conditions. The putative smORF datasets served as reference databases for subsequent optimized mass spectrometry analyses to identify *bona fide* miPs. All datasets presented herein contain detailed information on the location and sequence of each smORF and the corresponding identified miP, are searchable, and represent a valuable resource for future functional studies.

## 2. Methods

Detailed methods are presented in Supplementary Methods online.

### 2.1 Cell culture

Human umbilical vein endothelial cells were isolated and cultured as described previously,^30,31^ and used up to Passage 4. The use of human material in this study complies with the principles outlined in the Declaration of Helsinki (World Medical Association, 2013), and the isolation of endothelial cells was approved in written form by the ethics committee of the Goethe-University. All cells were negative for mycoplasma contamination. Cultured cells were kept in a humidified incubator at 37°C containing 5% CO_2_.

### 2.2 Transcoronary ablation of septal hypertrophy patient samples

Eight patients (characteristics summarized in Supplementary material online, *Table S17*) with hypertrophic obstructive cardiomyopathy who were undergoing transcoronary ablation of septal hypertrophy (TASH) were included in the study. The pre- and post-procedural management of these patients was as documented.^32^ In brief, diagnostic assessment was performed according to the current guidelines, based on severe symptoms, asymmetrical septal hypertrophy (>15 mm), systolic movement of the anterior mitral valve leaflet, and an intraventricular pressure gradient of 30 mmHg at rest and/or 50 mmHg after Valsalva manoeuvre. TASH was performed according to standard clinical practice. All patients provided written informed consent for their participation in the study according to the Declaration of Helsinki, and the ethics board of the state of Hessen, Germany, approved the study (FF 31/2010). Venous blood samples for miP detection were collected before (baseline) and four hours after induction of myocardial infarction using TASH. Samples were processed immediately, and serum was frozen at −80°C.

### 2.3 Human carotid artery samples

Human carotid tissue from the Munich Vascular Biobank^33^ was studied, and all patients had provided written and informed consent according to the Declaration of Helsinki. Tissue sampling was approved by the local ethics committee (Ethikkommission Klinikum rechts der Isar: 2799/10). Human carotid specimens were harvested during carotid endarterectomy. All patient data are summarized in Supplementary material online, *Table S18*.

### 2.4 Animals

Endothelial cell-specific RiboTag mice (EC-RiboTag) were generated by crossing endothelial cell-specific Cre-driver mice (Cdh5-CreERT2)^34^ with Ribo-Tag (*Rpl22*^HA/HA^)^29^ mice. The offspring were backcrossed with C57BL/6J mice (Charles River) for at least 8–10 generations. Both male and female mice were included in all studies. Cre-mediated recombination was induced in 8- to 9–week-old mice by intraperitoneal injections of tamoxifen (50 mg/kg/day dissolved in 50 µL Miglyol; Merck, Darmstadt, Germany) for 5 consecutive days. Animals were housed under a 12 h light–dark cycle with free access to water and a normal chow diet. One group of animals was sacrificed after deep anaesthetization with 180 mg/kg ketamine and 16 mg/kg xylazine, 10 days after the last tamoxifen injection. Thereafter, the thorax was opened, and intracardiac perfusion (through the left and right ventricles) was performed with 10 mL 100 μg/mL cycloheximide (Sigma-Aldrich, Darmstadt, Germany) in PBS. After perfusion, vessels and organs were harvested, snap frozen in liquid nitrogen and stored at −80°C until use. Male ApoE^−/−^ mice (8–10 weeks of age) were purchased from Charles River Laboratories (Sulzfeld, Germany). Accelerated endothelial activation and dysfunction were induced by partial ligation of the left carotid artery as described.^35,36^ Two or 7 days after ligation, animals were placed in an airtight anaesthesia box and the oxygen-isoflurane mixture was then flooded with >5% isoflurane via an isoflurane vaporizer. Animal death was confirmed by the complete cessation of breathing for at least 1 min and the absence of paw reflex responses. Vessels and organs were harvested, snap frozen in liquid nitrogen and stored at −80°C until processed. All animal experiments were performed in accordance with the Directive 2010/63/EU of the European Parliament on the protection of animals used for scientific purposes and approved by the Federal Authority for Animal Research at the Regierungspräsidium Darmstadt (Hessen, Germany) under study protocols B2/1102, B2/1187 and F28/42.

### 2.5 Statistics

The statistical tests used in each analysis are reported in the respective section of the Methods and Figure legends. Results are presented as mean ± standard error of mean (SEM). GraphPad Prism software (v. 10) was used to assess statistical significance. Differences between the two groups were compared by a two-tailed unpaired *t*-test. Experiments in which the effects of two variables were tested were analysed by two-way analysis of variance (ANOVA) followed by Šídák's multiple comparisons test. Differences were considered statistically significant when *P* < 0.05. Only exact significant *P-*values are reported.

## 3. Results

### 3.1 High-throughput proteogenomic identification of endothelial cell microproteins

**Figure 1:**
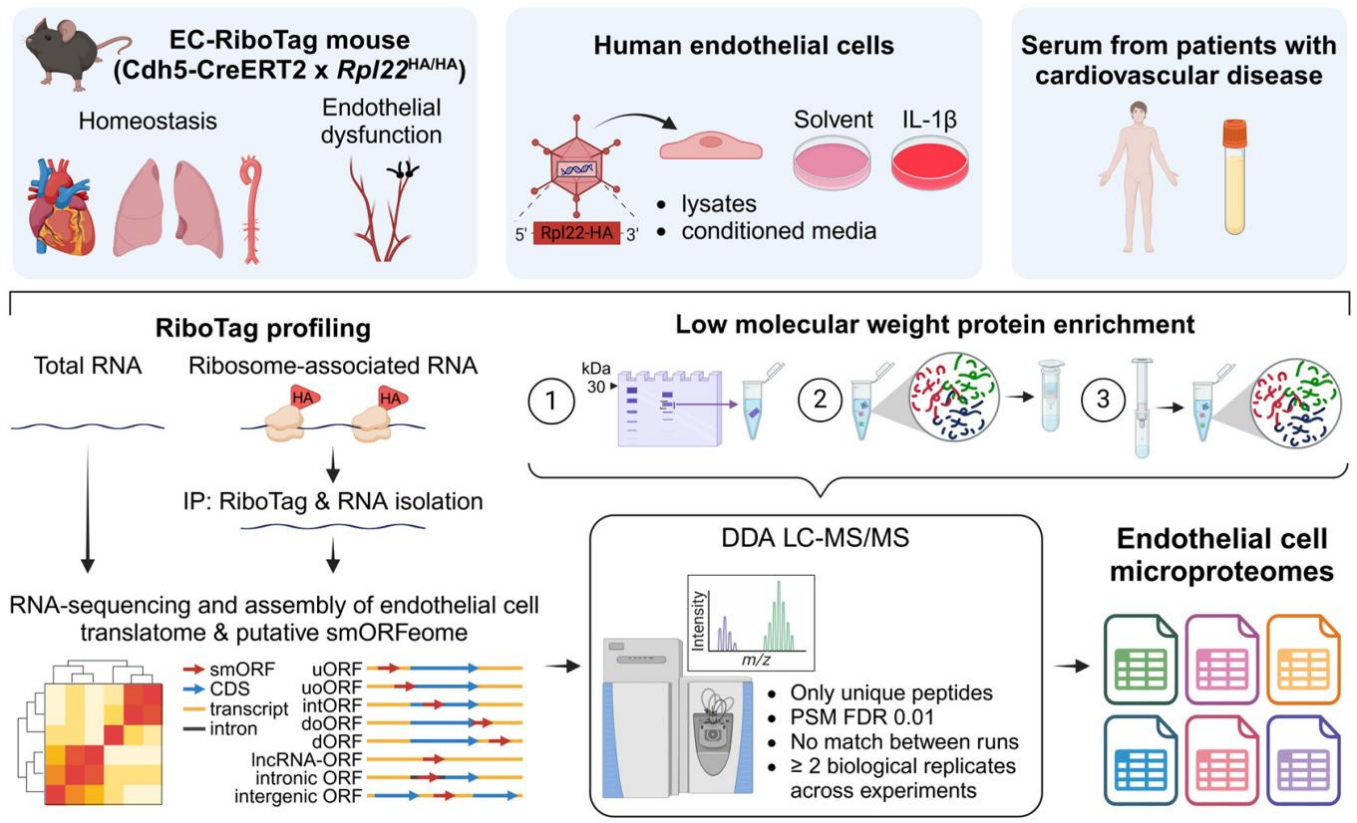
High-throughput proteogenomic identification of endothelial cell miPs. Tissues from endothelial cell (EC)-specific RiboTag mice as well as human endothelial cells expressing the RiboTag under homeostatic and inflammatory conditions were used to sequence total as well as ribosome-associated RNA to identify transcripts undergoing translation. The datasets were processed with bioinformatic pipelines to identify putative endothelial cell smORFs, which were then classified based on their genomic location relative to currently annotated transcripts/genes: uORF: upstream ORF, uoORF: upstream overlapping ORF, intORF: internal ORF, doORF: downstream overlapping ORF, dORF: downstream ORF, lncORF: ORF in transcript currently annotated as non-coding RNA, intronic ORF: ORF entirely contained within a reference intron, intergenic ORF: ORF in transcript from intergenic region, other: ORF that does not fall into any of the categories above (e.g. ORF does not overlap an annotated UTR, ORF starts upstream of an annotated UTR, ORF is partially intronic). The resulting endothelial smORFeome datasets were translated *in silico* and used to search mass spectrometry datasets generated from the same samples using an extensively optimized sample preparation protocol to enrich the low molecular weight proteome and maximize detection of miPs. Sample preparation before mass spectrometry included: 1) In gel trypsin digestion of proteins smaller than 30 kDa, 2) In solution digestion with Lys-C and trypsin and fractionation with high pH reversed-phase columns, and 3) Isolation of small proteins through reversed-phase C8 cartridges followed by in solution digestion with Lys-C and trypsin. This integrated proteogenomic analysis resulted in the identification of the endothelial cell microproteome, which consisted exclusively of currently unannotated peptides. Created in BioRender. Siragusa, M. (2026) https://BioRender.com/ sdbqyjy

To assess the endothelial cell-specific translation of smORFs into miPs in highly vascularized organs and blood vessels, we made use of the RiboTag mouse, which carries the ribosomal subunit Rpl22 allele with a floxed wild-type C-terminal exon, followed by an identical exon containing three haemagglutinin (HA) epitope copies inserted before the stop codon.^29^ The RiboTag was made inducible and endothelial cell-specific by crossing with Cdh5-CreERT2 mice (Cdh5-CreERT2*×Rpl22*^HA/HA^),^37^ so that following the application of tamoxifen, endothelial cells express the HA-tagged ribosomal subunit Rpl22, which integrates into actively translating polyribosomes. A similar approach was applied to cultured human endothelial cells transduced with adenoviruses to express the RiboTag. The affinity purification of tagged ribosomes using an HA-specific monoclonal antibody and subsequent sequencing of ribosome-associated RNAs compared to total RNAs enabled the study of alterations in the translation of endothelial cell-specific transcripts in tissues and in cultured cells. The resulting datasets were processed with a transcriptome assembler/ORF caller (MAPS) specifically designed to be used in conjunction with proteomics.^38^ The analysis was set to find putative unique smORFs with the following features: (i) 20–100 codons in length, (ii) starting with a canonical or near-cognate start codon preceded by a Kozak sequence, or if neither were found, ORFs downstream of an in-frame stop codon,^1,4,38,39^ and (iii) coding for peptides not listed in the UniProt reference database. The resulting putative smORFeome datasets were translated *in silico* and used together with the UniProt reference database to search mass spectrometry data generated from the same samples. The sample preparation protocol for proteomics was optimized and multiple methods were combined to enrich the low molecular mass proteome in tissue and cell samples to facilitate the detection of miPs by mass spectrometry (see Methods). In addition, extensive liquid chromatography–mass spectrometry fractionation and acquisition times maximized the chance of detecting unique peptides that matched the *in silico* translated smORFeome databases. Stringent identification filtration was performed using a Peptide-Spectrum Match False Discovery Rate of 0.01, a widely used approach for identifying miPs.^1,3,4,6,8,11^ Importantly, hits were considered to be *bona fide* miPs only when they were identified in at least two biologically independent samples. This information is important because in every mass spectrometry analysis, except the analysis of serum samples, where a direct comparison between before and after a surgical procedure in the same individuals was required, we set the parameter ‘match between runs’ to false (see Supplementary material online, Tables). This means that each miP peptide identified in one experiment was independent from whether or not the same peptide was detected in other biological replicates in that same experiment (*Figure 1*). Key to our approach was the use of sizable biological replicates for both the RNA sequencing and proteomic workflows. This integrated proteogenomic analysis resulted in the identification of microproteomes that excluded isoforms, as well as truncation and degradation products of previously annotated proteins and consisted exclusively of currently unannotated peptides.

### 3.2 The murine endothelial cell microproteome under homeostatic conditions

**Figure 2:**
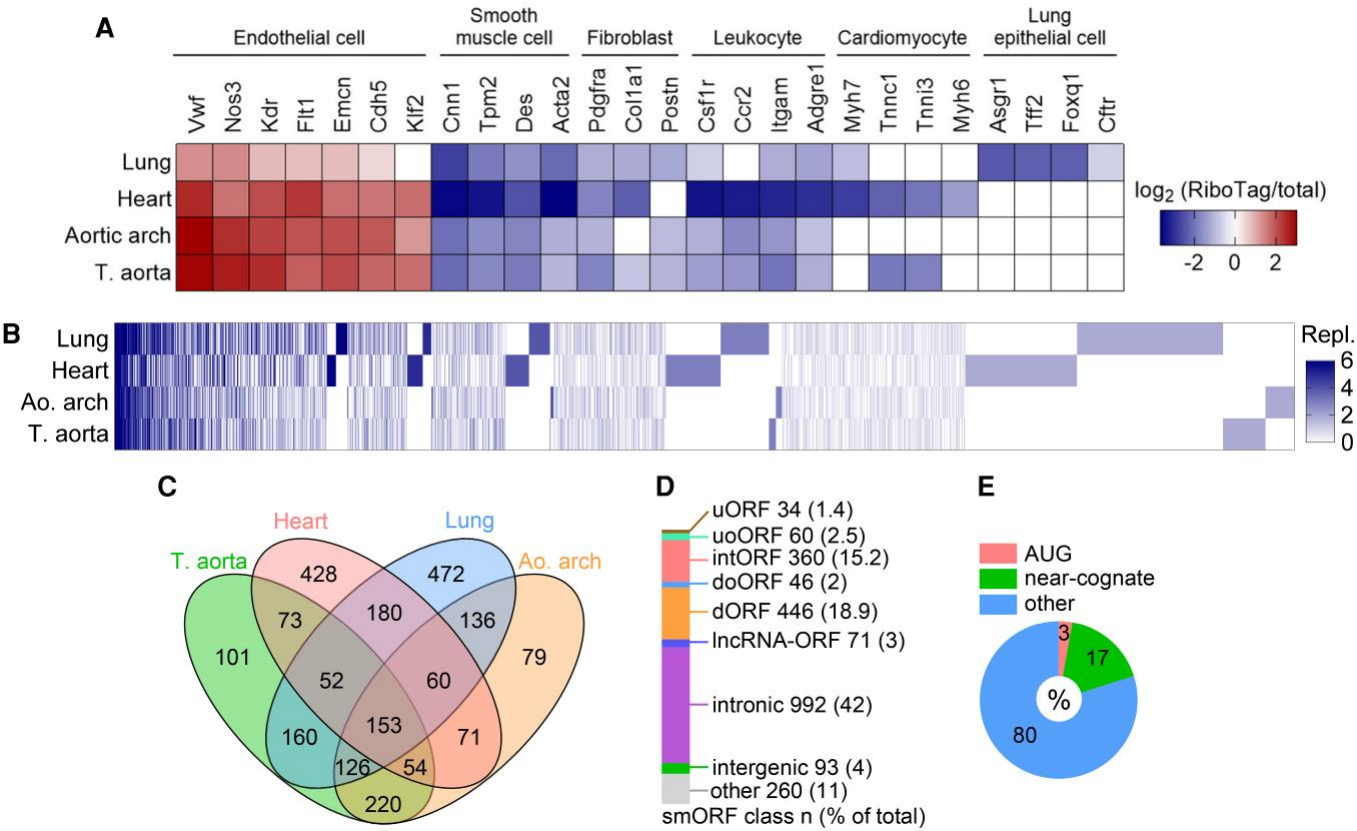
The murine endothelial cell microproteome under homeostatic conditions. A, Heat map demonstrating successful endothelial cell enrichment by expression of marker transcripts in tissues from EC-RiboTag mice (RiboTag RNA relative to total RNA from the same samples) FDR&lt;0.05 for all comparisons. B, Heat map showing the number of biological replicates in which each miP was detected by mass spectrometry in the indicated tissues. C, Number of murine endothelial cell miPs identified by mass spectrometry in one or more of the indicated tissues. D, Classification of smORFs encoding the 2365 endothelial cell miPs validated by mass spectrometry based on their genomic location and position relative to annotated transcripts/genes. E, Start codon usage of smORFs encoding the 2365 murine endothelial cell miPs validated at the proteomic level. *n* = 5 mice (lung); *n* = 6 mice (heart, aortic arch and thoracic aorta).

The pipeline described above was used to identify miPs expressed by native endothelial cells from RiboTag mice. Whole transcriptome analysis confirmed the effective enrichment of endothelial genes in RiboTag-associated transcripts compared to total transcripts from lung, heart, aortic arch and thoracic aorta (*Figure 2A*, Supplementary material online, *Table S1*). MAPS assembly identified 379 370 putative smORFs in 46 859 endothelial cell transcripts; including 25 794 from intergenic regions (see Supplementary material online, *Table S2*). Subsequent proteomic analyses using the *in silico* translation of this mouse smORF database concatenated to the UniProt reference database validated the expression of a total 2365 murine endothelial cell miPs identified in at least two biological replicates (Peptide-Spectrum Match False Discovery Rate: 0.01) (*Figure 2B* and *C*, Supplementary material online, *Tables S2* and *S3*). 54.3% of the miPs were common to two or more organs and the majority of miPs were encoded by unspliced smORFs (86.9% vs. 13.1% spliced smORFs), that is, entirely within one exon or intron of annotated protein coding transcripts. While 20.3% of the miPs were encoded by smORFs within the 5′ or 3′ untranslated regions (UTRs) of annotated protein coding transcripts (upstream ORFs or uORFs: 1.4%, downstream ORFs or dORFs: 18.9%), 19.8% partially (upstream/downstream overlapping uo/doORFs: 4.6%) or entirely (internal intORFs: 15.2%) overlapped the coding sequence of annotated protein coding transcripts, albeit in a different reading frame. A surprisingly large number of miPs (41.9%) were encoded by intronic smORFs. In line with previous reports,^6,7^ some miPs were derived from smORFs within transcripts annotated as long non-coding RNAs (lncRNA-ORFs: 3%), or regions annotated as intergenic (3.9%) (*Figure 2D*). Unexpectedly, only 2.8% of the smORFs encoding the identified miPs started with a canonical AUG start codon, 17.3% started with near-cognate codons, while the majority had non-canonical start sites (*Figure 2E*). The miPs identified had an average length of 53 ± 0.4 amino acids and a wide range of hydropathies (see Supplementary material online, *Figure S1A* and *B*).

### 3.3 Endothelial cell activation/inflammation alters smORF and miP expression *in vivo*

**Figure 3:**
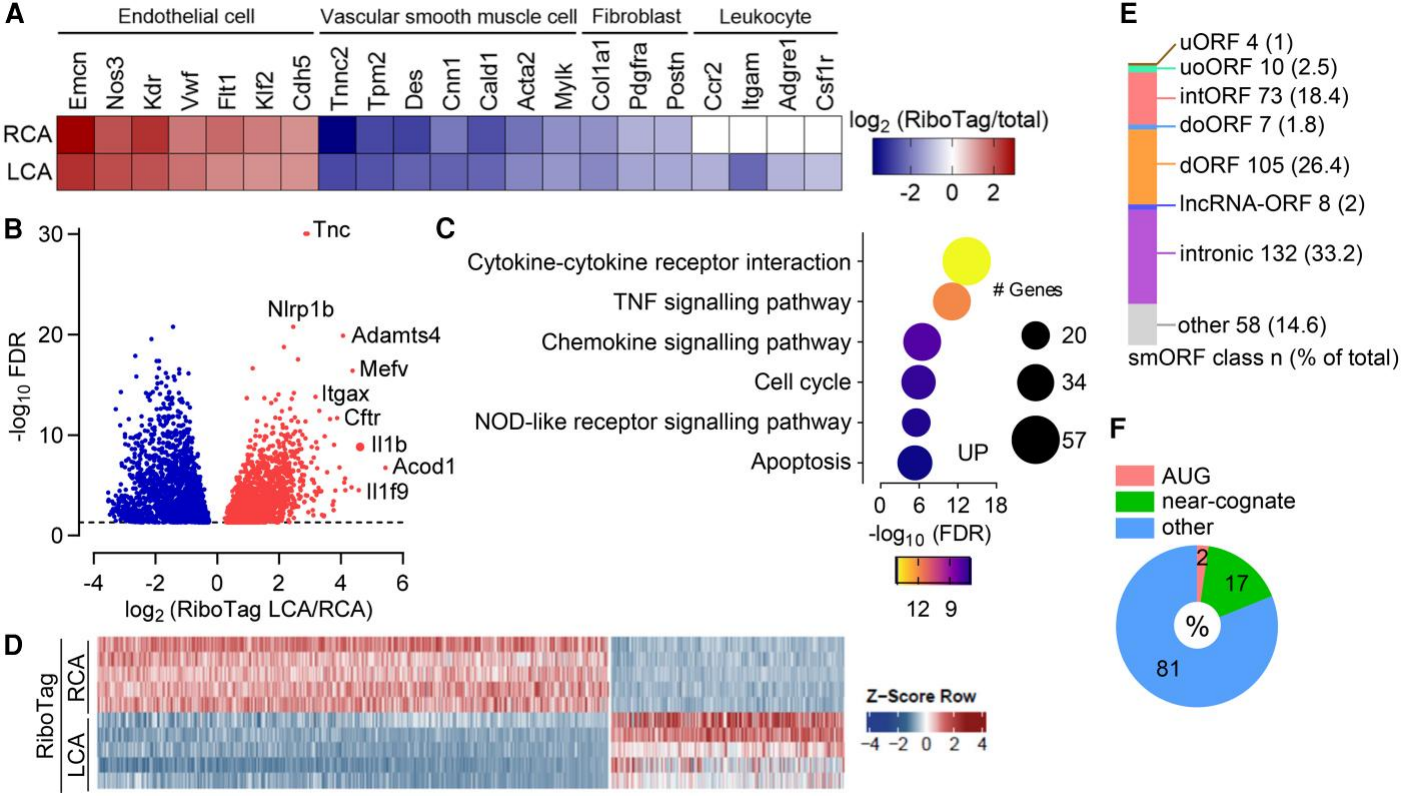
Effect of endothelial cell activation/inflammation on the endothelial cell microproteome *in vivo*. A, Heat map demonstrating successful endothelial cell enrichment of transcripts from left (LCA) and right (RCA) carotid arteries from EC-RiboTag mice 7 days after partial LCA ligation. Shown are RiboTag-associated transcripts relative to total RNA; *n* = 5 carotid artery samples with 5–7 mice/sample (FDR&lt;0.05 for all comparisons). B, Volcano plot depicting the upregulated (red) and downregulated (blue) RiboTag-associated transcripts in murine carotid arteries 7 days after partial LCA ligation. C, Gene set enrichment analysis (KEGG) of upregulated transcripts in LCA vs. RCA endothelial cells. D, Heat map showing relative expression Z-scores of the endothelial smORFs differentially regulated following partial LCA ligation; *n* = 5 carotid artery samples (5–7 mice/sample). E, Classification of smORFs encoding the 397 endothelial cell miPs verified by mass spectrometry, based on their genomic location relative to currently annotated transcripts/genes. F, Start codon usage of smORFs encoding the 397 murine endothelial cell miPs validated by mass spectrometry.

To study the effects of endothelial cell activation and inflammation on the expression of endothelial smORFs *in vivo*, endothelial cell-specific (EC)-RiboTag mice were made hypercholesterolaemic using a combination of the recombinant adeno-associated virus (rAAV)-mediated hepatic overexpression of proprotein convertase subtilisin/kexin type 9 and a high-cholesterol diet. These mice also underwent partial left carotid artery ligation to induce disturbed flow and accelerate atherogenesis.^35^ RiboTag-seq of carotid arteries 7 days post ligation, that is, at the peak of the inflammatory response and endothelial dysfunction,^40^ confirmed the enrichment of endothelial transcripts and the upregulation of genes characteristically associated with the inflammatory activation of the endothelium in the ligated left carotid artery, including IL-1β (*Figure 3A–C*, Supplementary material online, *Table S4*). The translation of 14 411 transcripts, containing a large number of putative smORFs, was significantly altered in ligated vs. non-ligated carotid arteries. Proteomic confirmation was obtained for 397 miPs (*Figure 3D*, Supplementary material online, *Tables S5* and *S6*). The features of the miP-encoding smORFs were similar to those of the murine endothelial smORFs/miPs identified in other murine organs under homeostatic conditions (*Figure 3E* and *F* and Supplementary material online, *Figure S1C* and *D*).

### 3.4 The human endothelial cell microproteome in homeostasis and inflammation

**Figure 4:**
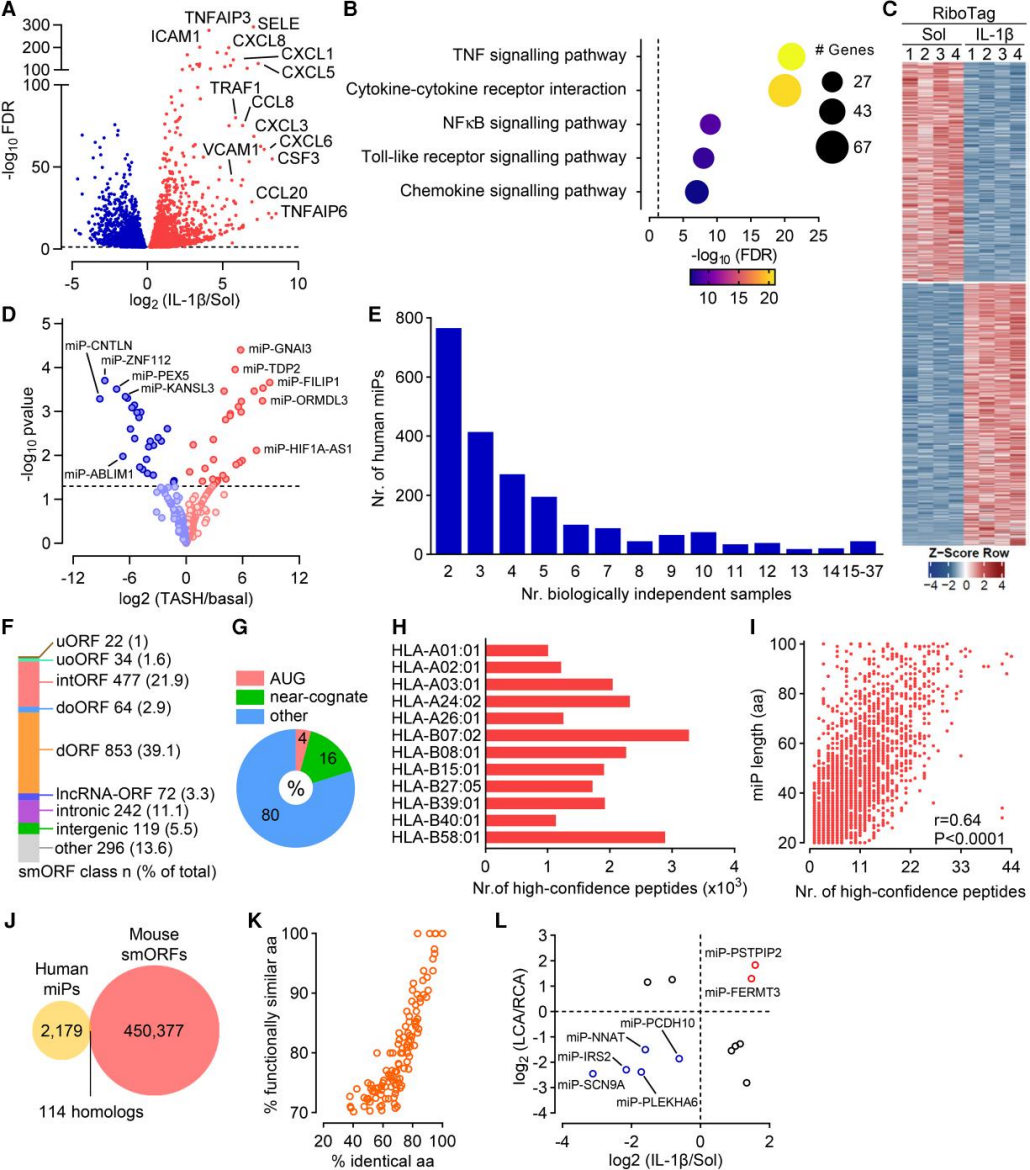
The human endothelial cell microproteome. A, Volcano plot showing RiboTag-associated transcripts in human endothelial cells upregulated (red) and downregulated (blue) following treatment with IL-1β; *n* = 4 independent cell batches. B, Enrichment analysis (KEGG) of transcripts upregulated by IL-1β. C, Heat map showing Z-scores of endothelial smORF-containing transcripts differentially expressed in solvent- and IL-1β-treated endothelial cells. D, Volcano plot showing miPs identified in the serum of patients who underwent TASH. Red = miPs increased after TASH; blue = miPs decreased after TASH; *n* = 8 individuals. The dashed line marks the significance threshold (P = 0.05). E, Number of biologically independent samples in which one or more unique peptides matching a miP were identified by mass spectrometry in all human datasets. F, Classification of smORFs encoding the 2179 endothelial cell miPs detected by mass spectrometry based on their genomic location relative to currently annotated transcripts/genes. G, Start codon usage of smORFs encoding the 2179 endothelial cell miPs validated by mass spectrometry. H, Number of high-confidence endothelial cell miP-derived peptide binders (length 8–12 aa) per HLA class I supertype. *(I*) Correlation between miP length and number of predicted high-confidence HLA class I peptide binders for each miP. J, Homology analysis between human miPs validated at the proteomic level and murine endothelial cell *in silico* translated smORFs. K, Scatter plot showing amino acid similarity between homologous human and mouse miPs. L, Scatter plot showing inflammation-induced alterations in the expression of homologous miPs in human (RiboTag IL-1β/solvent) and murine (RiboTag LCA/RCA) endothelial cells.

To study the human endothelial cell smORFeome, human endothelial cells were transduced with adenoviruses to express the RiboTag and treated with either solvent or IL-1β. The latter was chosen as it is the pro-inflammatory cytokine most upregulated in response to vascular damage and during atherogenesis,^41^ and its inhibition blunts the development of atherosclerosis in humans.^42^ Using this approach, 196 873 putative smORFs were annotated in 29 583 transcripts (including 11 718 from intergenic regions) under homeostatic conditions (see Supplementary material online, *Table S7*). IL-1β induced endothelial cell activation and elicited the upregulation of genes related to inflammation and cytokine/chemokine signalling pathways (*Figure 4A* and *B*, Supplementary material online, *Table S8*). In the same cells, our pipeline identified significant IL-1β-dependent alterations in the ribosome association of 11 695 transcripts (including 3174 from intergenic regions) containing 79 582 putative smORFs (*Figure 4C*, Supplementary material online, *Table S9*). Homeostatic and IL-1β-stimulated endothelial cells were also subjected to proteomic analyses, which led to the validation of 1365 distinct smORF-encoded miPs (see Supplementary material online, *Tables S7* and *S9*) with no comparable sequences listed in UniProt.

miPs can also be released from cells,^43,44^ and given the key role of the endothelium in mediating crosstalk between tissues and the circulation, we hypothesized that endothelial cells release miPs. It was possible to identify 607 distinct extracellular miPs by mass spectrometry in conditioned media from endothelial cells cultured under either homeostatic conditions or following stimulation with IL-1β (see Supplementary material online, *Table S10*). To detect circulating miPs and their alterations in response to injury in humans, we collected blood samples before and 4 h after TASH, a catheter interventional treatment for hypertrophic obstructive cardiomyopathy that decreases septal thickness.^45^ Using our endothelial cell database as a reference for mass spectrometry, we identified 347 miPs in human serum, 26 of which were significantly decreased and 31 significantly increased following the procedure (*Figure 4D*, Supplementary material online, *Table S11*).

Altogether, the proteogenomic approach provided evidence for the expression of 2179 novel miPs in human endothelial cells. These *bona fide* miPs were identified in at least two independent human samples across all proteomic searches (*Figure 4E*, Supplementary material online, *Table S12*). The majority of these miPs were encoded by unspliced smORFs (84.1% vs. 15.9% spliced smORFs). Roughly 40% of the miPs were encoded by uORFs or dORFs and 26.4% by uo/doORFs (4.5%) or intORFs (21.9%). The remaining miPs were derived from lncRNA-ORFs (3.3%), intronic (11.1%) or intergenic (5.5%) ORFs (*Figure 4F*). Only 4.2% of the smORFs had a canonical AUG start codon, 16% started with near-cognate codons, while the majority had non-canonical start sites (*Figure 4G*). The average length of human miPs was 48.1 ± 0.4 amino acids, and both hydrophilic and hydrophobic miPs were equally present (see Supplementary material online, *Figure S1E* and *F*).

Evidence often provided to support the existence as well as the biological relevance of miPs is the presentation of miP-derived peptides on HLA molecules.^9,16,46^ We therefore predicted the binding affinity of endothelial miP-derived peptides to 12 HLA class I supertypes using the state-of-the-art neural network-based NetMHCpan 4.1 database.^47^ Peptides derived from the majority of the miPs (98% i.e, 2133/2179 miPs) were predicted to bind HLA class I with high affinity (EL score ≤0.5) (*Figure 4H*, Supplementary material online, *Table S13*). There was a good correlation between the number of predicted high-confidence peptide binders and miP length (Pearson r: 0.64, *P* < 0.0001, *Figure 4I*). These findings suggest that the miPs identified are processed and presented as self-antigens by the cellular antigen presentation machinery.

Homology is traditionally considered indicative of functional relevance. We therefore performed a homology analysis between the 2179 human miPs that had been validated by mass spectrometry and the 450 377 putative mouse smORFs annotated across experiments. Homologous sequences were considered those that shared >70% functionally similar amino acids, that is, similar polarity, hydrophilicity or charge features, and covering >80% of each human/murine peptide sequence. We found 114 homologous sequences (96 distinct human miPs with one or more mouse homologues), which is in line with recent reports,^3,7,11,48–50^ and is consistent with the idea that smORFs may have evolved only recently (*Figure 4J* and *K*, Supplementary material online, *Table S14*). To identify homologous miPs with similar regulation in humans and mice, we compared the changes in expression of the corresponding smORFs under inflammatory conditions. This approach identified 7 homologous miPs that were similarly regulated in both human and murine samples and that were encoded by smORFs partially or entirely overlapping the same transcript in both species (*Figure 4L*).

### 3.5 Expression of endogenous miP-PSTPIP2 *in vitro* and in murine and human arteries

**Figure 5:**
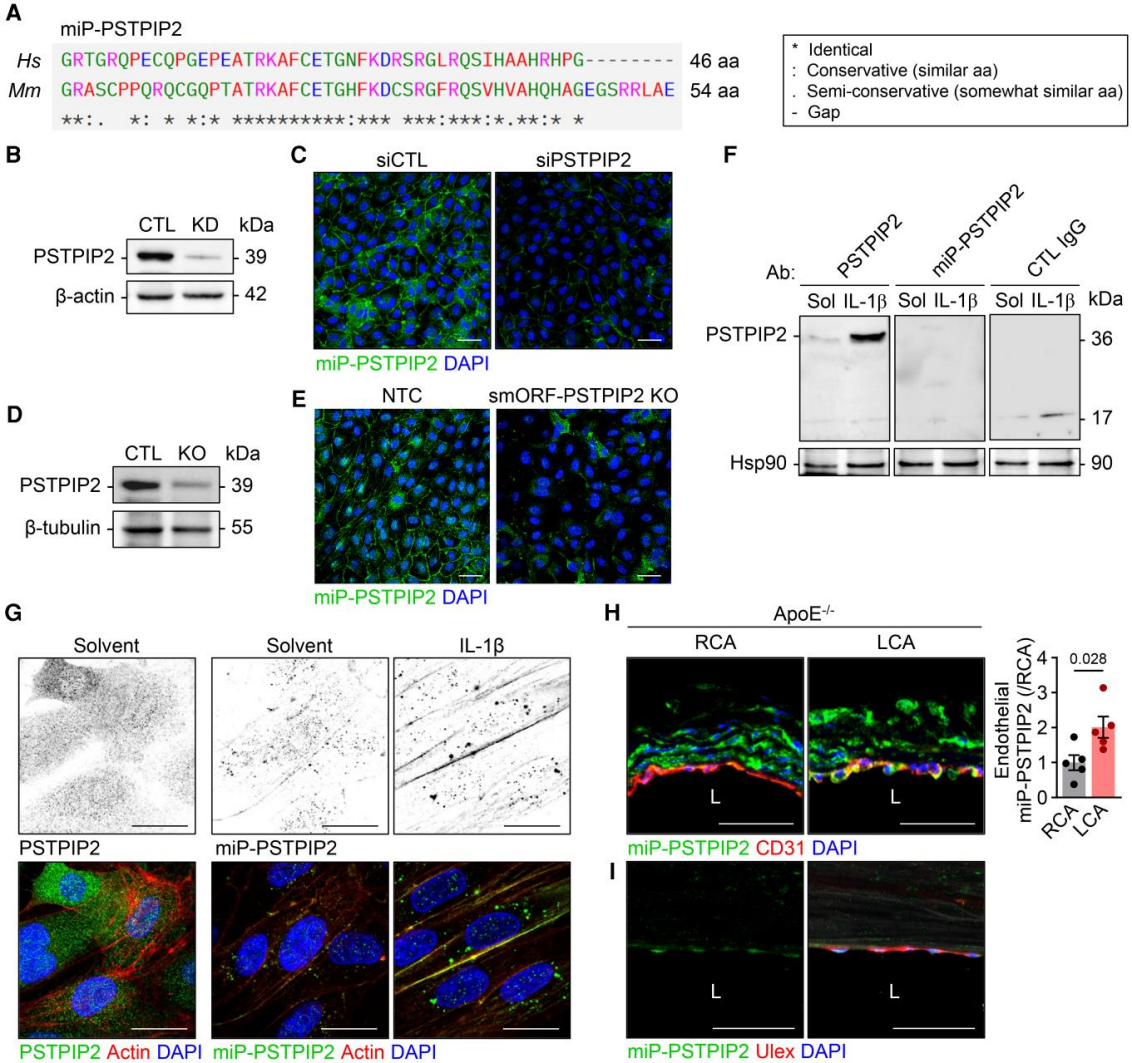
Expression of miP-PSTPIP2 in human and mouse. A, Sequence alignment between human and mouse miP-PSTPIP2, generated with Clustal Omega. B-C, Western blot showing PSTPIP2 (*B*) and representative confocal images showing miP-PSTPIP2 levels (custom antibody) (*C*) in human endothelial cells 48 h after transfection with siRNA targeting PSTPIP2 (KD) or a control siRNA (CTL). Nuclei were stained with DAPI. Similar results were obtained in 4 additional independent cell batches. Scale bar: 20 µm. *D*-*E*, Western blot showing PSTPIP2 (*D*) and representative confocal images showing miP-PSTPIP2 levels (custom antibody) (*E*) in human endothelial cells after lentiviral transduction with a CRISPR non-targeting control gRNA (NTC) or a gRNA targeting smORF-PSTPIP2 (KO). Nuclei were stained with DAPI. Similar results were obtained in 3 additional independent cell batches. Scale bars: 20 µm. F, Western blot demonstrating PSTPIP2 expression in solvent (Sol)- and IL-1β-treated human endothelial cells, along with the reactivity of the custom antibody raised against miP-PSTPIP2 or a control (CTL) IgG using identical protein samples; similar results were obtained in 3 additional independent cell batches. G, Representative confocal images showing PSTPIP2 or miP-PSTPIP2 and Actin (phalloidin) in solvent (Sol)- and IL-1β-treated human endothelial cells. Nuclei were stained with DAPI. Similar results were obtained in 3 additional independent cell batches. Scale bar: 10 µm. H, Representative confocal images and quantification of miP-PSTPIP2 in CD31 + endothelial cells in the non-ligated right (RCA) and ligated left (LCA) carotid arteries from ApoE-/− mice 2 days after ligation. L = lumen; *n* = 5 mice (two-tailed unpaired *t*-test). Scale bar: 50 µm. I, Representative confocal images showing miP-PSTPIP2 in endothelial cells (labelled with Ulex) in a human advanced carotid artery atherosclerotic plaque. L = artery lumen. Similar images obtained in samples from 10 additional patients. Scale bar: 50 µm.

To confirm the validity of our approach, we selected a miP encoded by a smORF within the transcript for proline-serine-threonine phosphatase-interacting protein 2 (*PSTPIP2*), which we refer to as miP-PSTPIP2. The smORF was the hit most upregulated by inflammation in murine and human cells and possessed a non-canonical start site. miP-PSTPIP2 was identified by mass spectrometry nine times in 12 samples from six biologically independent cell batches (*Figure 5A*). To visualize native miP-PSTPIP2, a custom polyclonal antibody was generated. An ELISA against the miP-PSTPIP2 peptide used for host immunization demonstrated that the affinity-purified antibody consisted of a rich antibody population able to detect the miP-PSTPIP2 antigen with high specificity (see Supplementary material online, *Figure S2*). As miP-PSTPIP2 is encoded by an intORF, siRNA-mediated knockdown or CRISPR/Cas9-mediated knockout of the *PSTPIP2* transcript in human endothelial cells was performed to further validate the specificity of the antibody. These approaches resulted in the depletion of both the PSTPIP2 protein and the miP-PSTPIP2 (*Figure 5B–E*). *PSTPIP2* was upregulated under inflammatory conditions *in vivo* (see Supplementary material online, *Table S4*) and *in vitro* (see Supplementary material online, *Table S8*) and treating endothelial cells with IL-1β increased the levels of PSTPIP2 protein (*Figure 5F*). The amino acid sequences of PSTPIP2 and miP-PSTPIP2 are distinct (different reading frames) and probing the same samples with the PSTPIP2 and miP-PSTPIP2 antibodies did not result in overlapping signals. Next, confocal microscopy was used to detect different expression patterns of the PSTPIP2 protein and miP-PSTPIP2 in human endothelial cells (*Figure 5G*). Endogenous miP-PSTPIP2 was localized to actin-rich domains, including the cell membrane, cytoskeleton, cytosolic vesicles and the nucleus. Consistent with the results of the proteogenomic pipeline, miP-PSTPIP2 expression was increased by treating human endothelial cells with IL-1β. The expression of miP-PSTPIP2 was also increased in ligated carotid arteries from ApoE^−/−^ mice fed a high-fat diet, a second model of hypercholesterolaemia and disturbed flow-induced endothelial cell activation (*Figure 5H*). Notably, the miP was also detectable in the endothelium of human carotid atherosclerotic plaques (*Figure 5I*, Supplementary material online, *Figure S3*). Thus, although detection of the anti-miP antibody was not suitable for immunoblotting, it provided consistent and reproducible results by immunohistochemistry.

### 3.6 Endothelial cell miPs modulate cell proliferation and survival

To identify human miPs that play a pivotal role in endothelial cell growth and survival, we designed a comprehensive CRISPR library targeting the smORFs encoding *bona fide* miPs that had been validated by mass spectrometry. Given that the majority of the smORFs targeted overlapped completely or partially with annotated genes, a strategy was developed to distinguish the effects of smORF perturbation from those of the overlapping genes. Up to three distinct guide RNAs (gRNAs) were designed for each smORF and another three gRNAs for different positions of the corresponding overlapping gene. Overall, the library consisted of 5818 gRNAs targeting 2793 smORFs and 6681 gRNAs targeting 2107 genes plus 94 intergenic regions that were more than 1 kb from the nearest intergenic smORF (see Supplementary material online, *Table S15*). This approach ensured that any effects observed could be attributed to the targeted smORFs, the overlapping genes or both.

**Figure 6:**
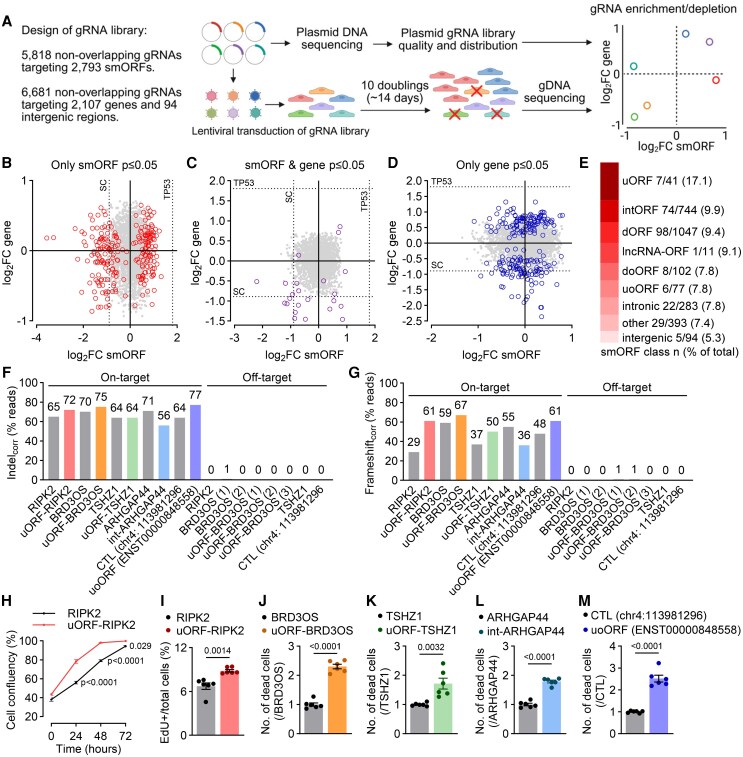
Endothelial cell miPs modulate cell proliferation and survival. A, Experimental design of the CRISPR screening to identify essential smORFs in the human endothelial cell line EA.hy926. B-D, Scatter plots showing the abundance of gRNAs targeting smORFs and gRNAs targeting the CDS of corresponding genes vs. the initial plasmid gRNA library distribution. Positive values indicate that targeting the smORF and/or gene increased cell growth (increased gRNA abundance), while negative values indicate decreased cell viability (reduced gRNA abundance). Red circles (*B*) depict smORF knockout-specific effects i.e. only gRNAs targeting the smORF but not those targeting the CDS of the parent gene, were significantly enriched or depleted. Purple circles (*C*) depict significant effects of targeting smORFs and genes at the same genomic location i.e. gRNAs targeting both smORFs and CDS of the parent genes were significantly enriched or depleted. Blue circles (*D*) depict gene knockout-specific effects i.e. only gRNAs targeting the CDS of a gene were significantly altered. Grey dots indicate smORF/gene pairs that had no effect on cell growth or viability. Significance threshold P = 0.05; log_2_FC threshold: ±0.138. Dotted lines indicate the effect (log_2_FC value) of two control gRNAs: SuperCutter (SC), which reduced endothelial cell viability and TP53, which increased endothelial cell growth. E, Frequency of smORF class knockout-specific effects relative to the total number of gRNAs targeting the respective class in the gRNA library. F-G, Percentage of reads containing indels (*F*) and indel-induced frameshifts (*G*) among all target reads, as determined by amplicon-based next-generation sequencing of on-target and predicted off-target sites in EA.hy926 cells following CRISPR/Cas9-mediated targeting of the indicated smORF/gene pairs from the high-throughput CRISPR screen shown in D-E. Values were background-corrected by subtracting the indel or frameshift rate observed in wild-type EA.hy926 cells due to sequencing and alignment artifacts at the ends of the amplicons. H-I, Confluency (*H*) and EdU incorporation (*I*) as a surrogates for proliferation in EA.hy926 cells following CRISPR/Cas9-mediated targeting of uORF-RIPK2 or RIPK2. *n* = 6 independent experiments (H: two-way ANOVA and Šídák's multiple comparisons test; I: two-tailed unpaired *t*-test). J-M, Propidium iodide-positive EA.hy926 cells following CRISPR/Cas9-mediated targeting of the indicated smORFs or the CDS of the corresponding genes. Data acquired 11 days after lentiviral transduction and 5 days after incubation with propidium iodide. *n* = 6 independent experiments (two-tailed unpaired *t*-test).

Guide RNAs were cloned to create a lentiviral library that was subsequently transduced into the EA.hy926 endothelial cell line. The abundance of gRNAs was measured by deep sequencing after 10 cell doublings and correlated to the initial library frequency to assess the impact of each gRNA on endothelial cell proliferation and viability. An increase in the abundance of a specific gRNA suggested that depletion of the targeted smORF/miP or gene/protein induced cell growth. Conversely, a decrease indicated that the targeted smORF/miP or gene/protein was essential for cell survival (*Figure 6A*). Controls included gRNAs targeting essential genes, for example, PCNA, POLR2L, RPS11 and RPS19 or highly repetitive elements in the genome (SuperCutter) to cause DNA damage, all of which resulted in loss of cell viability (see Supplementary material online, *Figure S4*). Guide RNAs targeting the tumour suppressor p53, in contrast, enhanced cell growth. In these experiments, a smORF-dependent effect was identified when gRNAs targeting the smORF elicited a response, while gRNAs targeting the gene or control intergenic region at the same genomic location had no effect. Using this approach, we found 116 smORFs that significantly increased endothelial cell proliferation when targeted, and 126 smORFs that reduced cell viability (*Figure 6B*, Supplementary material online, *Table S16*). In 15 cases, similar effects were achieved by targeting either the miP or the protein. For eight of the smORFs/genes, we observed opposite effects on proliferation and viability (*Figure 6C*). Additionally, we identified 224 genes whose deletion impacted cell function without there being any effect of targeting the overlapping smORFs (*Figure 6D*). Overall, the knockout of smORFs classified as uORFs more frequently affected cell growth or viability than other smORF classes (*Figure 6E*), which is consistent with a recent report in medulloblastoma cells.^10^ Next, we examined the impact of direct CRISPR/Cas9-mediated targeting of five smORF/gene pairs on cell proliferation by measuring confluency and EdU incorporation or cell death by assessing positivity to propidium iodide. On-target indel and frameshift formation and the absence of detectable mutations at predicted off-target sites were confirmed by amplicon-based next-generation sequencing. (*Figure 6F* and *G*, Supplementary material online, *Table S15*). In line with the results of the high-throughput screen, targeting uORF-RIPK2 increased cell proliferation, while targeting the parent gene, that is, *RIPK2* did not (*Figure 6H* and *I*). In contrast, targeting uORF-BRD3OS, uORF-TSHZ1, intORF-ARHGAP44 or the uoORF of the new transcript ENST00000848558 induced cell death, while targeting the parent gene did not (*Figure 6J–M*). These findings validated the results of the high-throughput CRISPR screen.

## 4. Discussion

Alterations in endothelial cell gene expression and function underlie cardiovascular disease. The discovery that endothelial cells express large numbers of miPs, and that the microproteome differs between cells from different organs as well as under control and pro-inflammatory conditions, hints at the importance of the cellular microenvironment in determining miP expression. Our data also highlights the potential significance of these molecules in the autocrine and paracrine regulation of vascular homeostasis and pathophysiology.

In this study, we applied a proteogenomic pipeline that combined RiboTag-sequencing with multiple approaches to enrich the low molecular mass proteome to facilitate the endothelial cell-specific identification of a large number of unannotated smORFs and their encoded miPs. By using the RiboTag, we took a step forward from transcriptomic-based approaches and focused on cell-type-specific translation to generate a list of putative smORFs, which served as a searchable database for subsequent proteomic validation studies. These candidates included smORFs with canonical as well as alternative start codons and smORFs overlapping previously annotated coding sequences (intORFs), which are difficult to annotate unambiguously using traditional ribosome profiling.^16,17^ The goal of our work was not to generate a stand-alone atlas of translated smORFs, but to cast a maximally diverse net to be used for validation of miP expression by mass spectrometry and followed up in future functional studies. Using this approach, we validated the presence of 2739 distinct endothelial cell miPs in murine tissues and 2179 distinct miPs in human endothelial cells and human serum samples. Approximately 20% of the corresponding smORFs possessed a canonical or near-cognate start codon, but the majority of validated miPs resulted from the translation of smORFs in a different reading frame relative to the currently annotated coding sequence. This was not entirely surprising, as previous studies had already provided strong evidence that smORF translation can be initiated at non-canonical start sites.^1,9,12,51,52^ The high number of smORFs possessing such start sites was, however, unexpected. While the exact start site is currently difficult to pinpoint, it is likely to be downstream the start site annotated by the MAPS assembler. Whether the translation of these sequences reflects leaky ribosome scanning, poorly understood ribosome recycling mechanisms,^51–53^ or changes in cell metabolism,^54^ remains to be determined.

The number of miPs identified in our study exceeds those in previous reports in other cells/tissues, but is feasible considering that ribosome profiling studies currently suggest the translation of ∼28 300 smORFs.^16,55^ We also detected a large number of miPs in human serum, and a change in the circulating miP profile following the TASH procedure. It should be noted that the identification of circulating miPs in human serum was performed using the endothelial smORFeome as a reference, but some of the detected miPs may also originate from other cell types rather than being exclusively endothelial-derived. Circulating miPs are of particular interest as, in addition to being potential new biomarkers of disease, they may act in a paracrine fashion to exert effects in receptive cells and tissues. Given their small size, effector miPs are likely to exhibit high druggability, with potential exploitation for the treatment of cardiovascular disease.

Additional supporting evidence for the identification of *bona fide* miPs in our study is that nearly all human miPs identified harboured peptides predicted to bind with high affinity to HLA class I, acting as naturally occurring auto-antigens. This is in line with other studies that demonstrated that peptides derived from non-canonical miPs could be detected in HLA immunopeptidomes.^9,16,46,56^ This finding was recently confirmed for 24.6% of 7264 GENCODE non-canonical Ribo-seq ORFs searched against ∼240 million mass spectrometry spectra aggregated within the Human HLA PeptideAtlas 2023-11 build.^57^ In the same study, there was also a strong concordance between HLA binding prediction algorithms and actual detection of peptides across public HLA immunopeptidomics datasets.

Unlike the relatively high homology that is frequently seen between proteins in different species, miPs only seem to be well conserved among primates.^11,49,58^ Our homology analyses revealed that largely different sets of miPs are expressed by humans and mice, which may imply that smORFs and their encoded miPs may represent ‘young’ genes/proteins and an evolutionary step distinguishing primates from other species. Consequently, studies with standard laboratory animals may be of limited use in characterizing specific human miPs. However, we did identify 114 mass spectrometry-validated human miPs with one or more homologues in mice. Among the miPs that exhibited consistent alterations in response to inflammation was miP-PSTPIP2, for which a custom antibody was successfully generated to visualize its expression and localization in endothelial cells. The miP was identified in IL-1β-treated endothelial cells, and consistent with this, elevated levels of miP-PSTPIP2 were detected in murine carotid arteries during the early stages of atherosclerosis, as well as in vessels from patients with advanced disease. While our data suggests a possible role for miP-PSTPIP2 in human atherosclerosis, future investigations are required to elucidate its functional relevance in endothelial cells. Such studies must consider that the smORF encoding miP-PSTPIP2 is an intORF, and while its sequence overlaps with that of *PSTPIP2*, it is in an alternative reading frame. Consequently, conventional knockdown approaches are likely to affect both the canonical protein and the miP. A more effective strategy would involve targeted gene editing to introduce a premature stop codon specifically within the smORF, while preserving the integrity of the main ORF. Alternatively, selective PSTPIP2 knockdown followed by re-expression of either PSTPIP2 or miP-PSTPIP2 should help dissect the specific role of the miP in endothelial cells. Lastly, given that miP-PSTPIP2 expression is upregulated under inflammatory conditions, controlled overexpression studies would provide valuable insights into its contribution to endothelial responses during inflammation and vascular dysfunction.

Working out the function and potential pathological relevance of the different miPs is a task for the future, but the high-throughput CRISPR/Cas9 screen, along with the subsequent validation experiments used here, enabled the identification of subsets of miPs that were required for endothelial cell proliferation and survival. This does not imply that the remaining miPs lack biological function but rather reflects the inherent limitation of the current screen, which was restricted to assessing only two quantifiable readouts. Future studies should explore the potential roles of miPs in a broader range of biological processes that are essential for endothelial function. Furthermore, although many of these miPs are expressed and detectable, their functional relevance may only manifest under specific stress conditions or environmental stimuli that were not captured within the scope of the present validation screen.

Taken together, the discovery that endothelial cells express large numbers of miPs, and that the endothelial microproteome varies across organs as well as under homeostatic and pro-inflammatory conditions hints at the importance of the cellular microenvironment in shaping miP expression. Furthermore, our findings highlight the potential significance of miPs in the autocrine and paracrine regulation of vascular homeostasis and pathophysiology. The generated miP datasets represent a treasure trove for studies assessing novel players in vascular physiology and disease mechanisms.

Translational perspective This study employed a high-throughput proteogenomic strategy to identify thousands of previously unannotated miPs in endothelial cells *in vitro* and *in vivo*. Endothelial cell miP expression was dynamically regulated under inflammatory conditions, suggesting a functional role in pathways implicated in the initiation and progression of vascular disease. Notably, the detection of miPs in the circulation of patients with cardiovascular disease indicates that they may participate in autocrine and/or paracrine signalling. Collectively, these findings highlight endothelial cell miPs as potential biomarkers of vascular health and as promising novel therapeutic targets for inflammation-driven vascular disease.

## Supplementary Material

cvag110_Supplementary_Data

## Data Availability

Further information and requests for resources and reagents should be directed to and will be fulfilled by the lead contact Mauro Siragusa (Siragusa@vrc.uni-frankfurt.de). The RiboTag recombinant adenovirus generated in this study will be made available from the lead contact upon request. Code is available without restrictions upon request to the lead contact. Data that support the findings of this study are available as part of the manuscript and the Supplemental Tables. Raw data can be accessed at the following repositories: NGS data: https://www.ncbi.nlm.nih.gov/geo/query/acc.cgi?acc=GSE195453 Mass spectrometry-based proteomics data: ProteomeXchange Consortium via the PRIDE^59^ or MassIVE partner repositories (http://proteomecentral.proteomexchange.org) with the following identifiers: Human endothelial cell microproteome—homeostasis and IL-1β: PXD031328 Released human endothelial cell miPs: PXD032004 Microproteome serum TASH patients: PXD032005 Murine endothelial cell microproteome—organs EC-RiboTag mouse: PXD032109 Murine endothelial cell microproteome—RCA vs. LCA EC-RiboTag mouse: PXD031363
