# Supplementary material for: Identification of unannotated microproteins involved in endothelial cell homeostasis, dysfunction, and vascular disease
Source: Cardiovasc Res. 2026 May 19;122(9):1257–70. doi: 10.1093/cvr/cvag110 (PMC13307568; doi:10.1093/cvr/cvag110)
Supplement: cvag110_Supplementary_Data [file cvag110_supplementary_data.zip › Siragusa et al_Suppl figures and figure legends_R1.pdf]

Identification of unannotated microproteins involved in endothelial cell homeostasis, dysfunction and vascular disease.

Siragusa M. et al.

Supplementary Figures

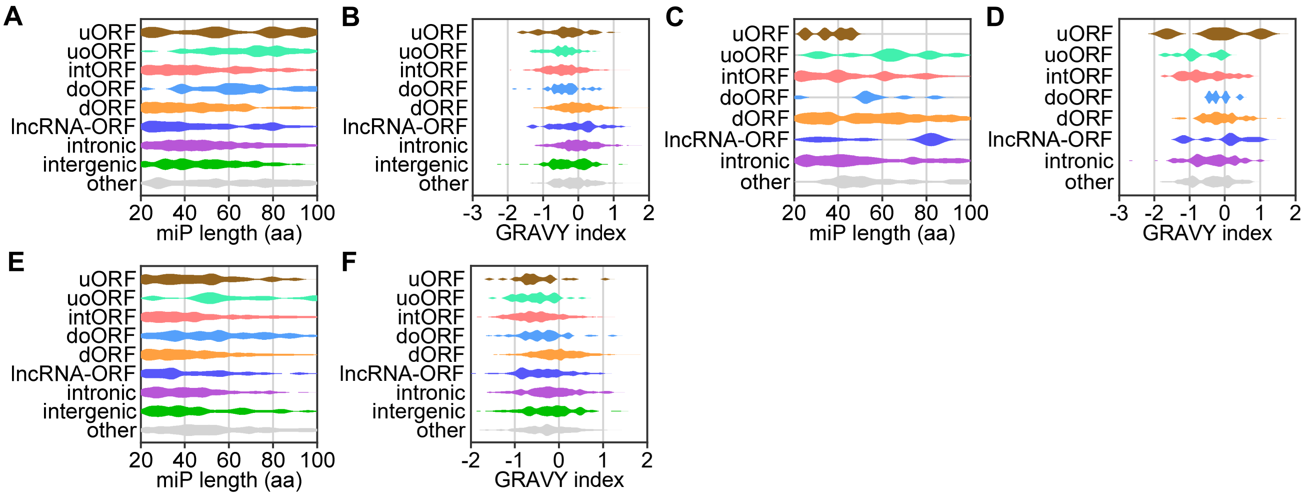

Figure S1. Length and hydropathy of human and murine endothelial cell miPs

A and **B,** Length (A) and hydropathy (GRAVY index) (B) of the 2,365 murine endothelial cell miPs (from lung, heart, aortic arch and thoracic aorta) validated by mass spectrometry. **C** and **D,** Length (C) and hydropathy (GRAVY index) (D) of the 397 murine carotid artery endothelial cell miPs validated by mass spectrometry. **E** and **F,** Length (E) and hydropathy (GRAVY index) (F) of the 2,179 human endothelial cell miPs validated by mass spectrometry.

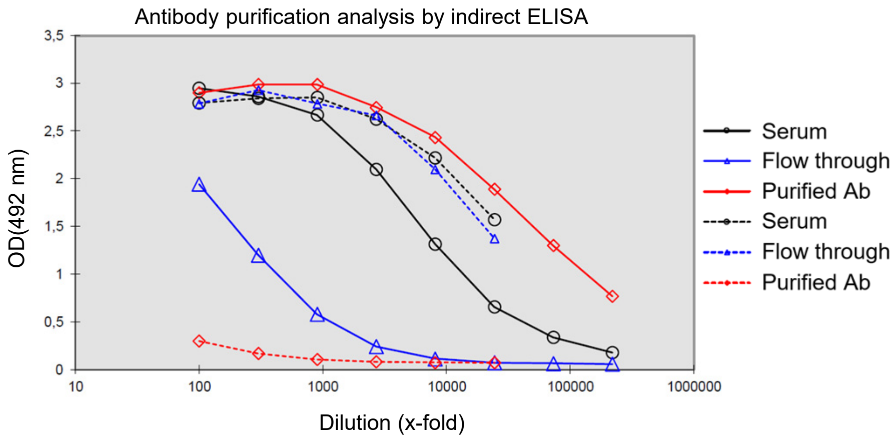

Figure S2. Validation of a custom antibody against miP-PSTPIP2.

Purification analysis by indirect ELISA of the custom antibody raised against miP-PSTPIP2 and used in this study. The antigen (continuous lines) or the carrier protein Keyhole Limpet Hemocyanin (KLH) (discontinuous lines) were coated into the wells of an ELISA plate and tested with increasing dilutions of either the serum from a rabbit immunized with the miP-PSTPIP2 peptide before affinity purification (black), the purified antibody (red) or the flow through (blue). The curves generated by the reaction of serum or the affinity-purified antibody with the miP-PSTPIP2 antigen (black and red continuous line, respectively) have a sigmoidal shape indicative of a rich antibody population that detects the miP-PSTPIP2 antigen with high specificity even if highly diluted. The curve related to the flow through after purification (blue continuous line) has an hyperbolic shape indicative of low concentration or affinity, as expected. The curve generated by the reaction of the purified antibody against the carrier protein KLH is flat, which demonstrates that the antibody recognizes specifically the miP-PSTPIP2 antigen, but not the KLH carrier.

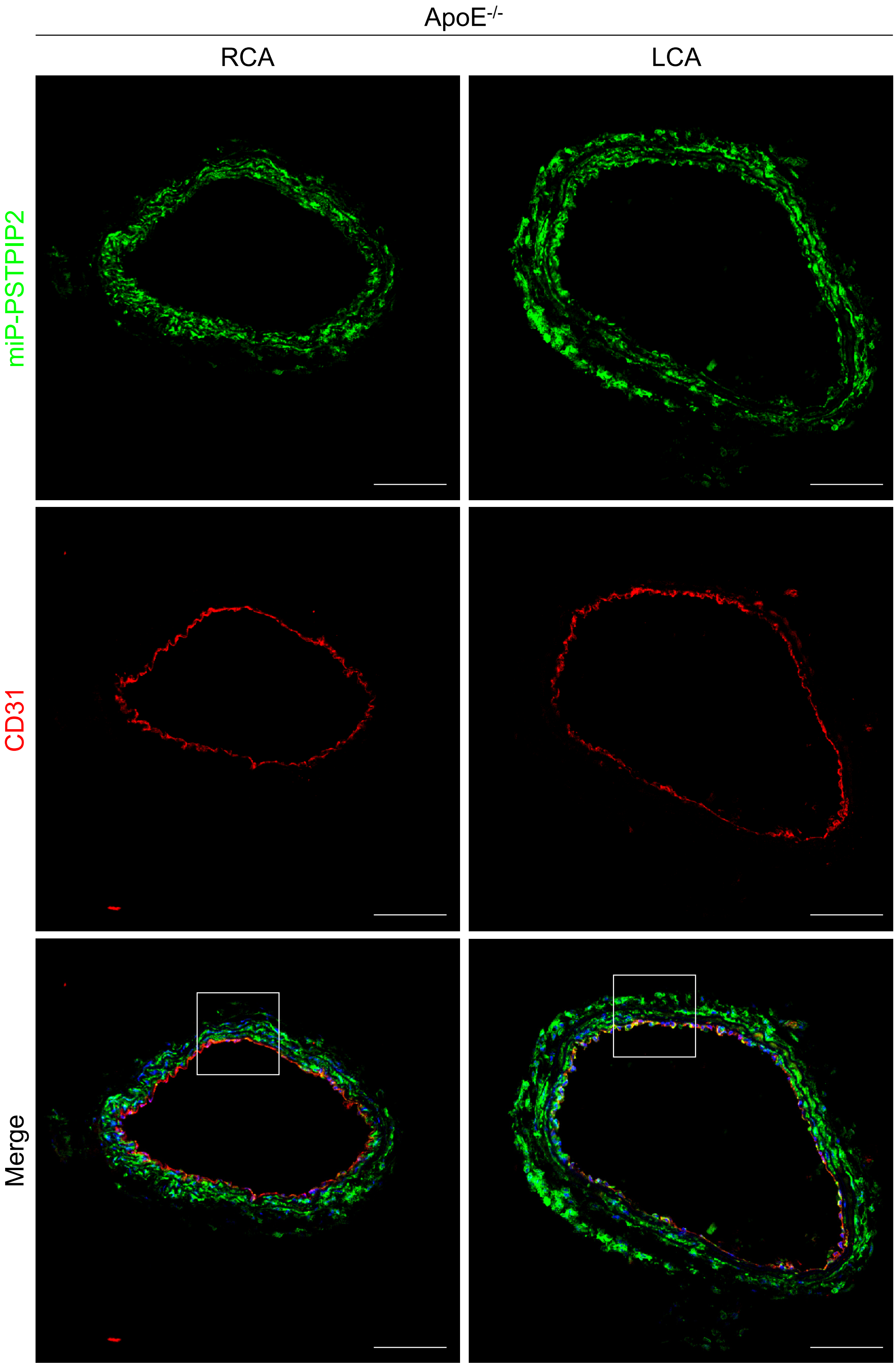

Figure S3. The expression of miP-PSTPIP2 is increased in the inflamed endothelium *in vivo*.

Representative confocal images of the expression of miP-PSTPIP2 in the CD31+ endothelium of non-ligated right (RCA) and ligated left (LCA) carotid arteries from ApoE$^{-/-}$ mice 2 days after ligation. The white squares in the merge images mark the areas presented in Fig. 5F. Comparable results were obtained in samples from 4 additional mice. Scale bar: 100 µm.

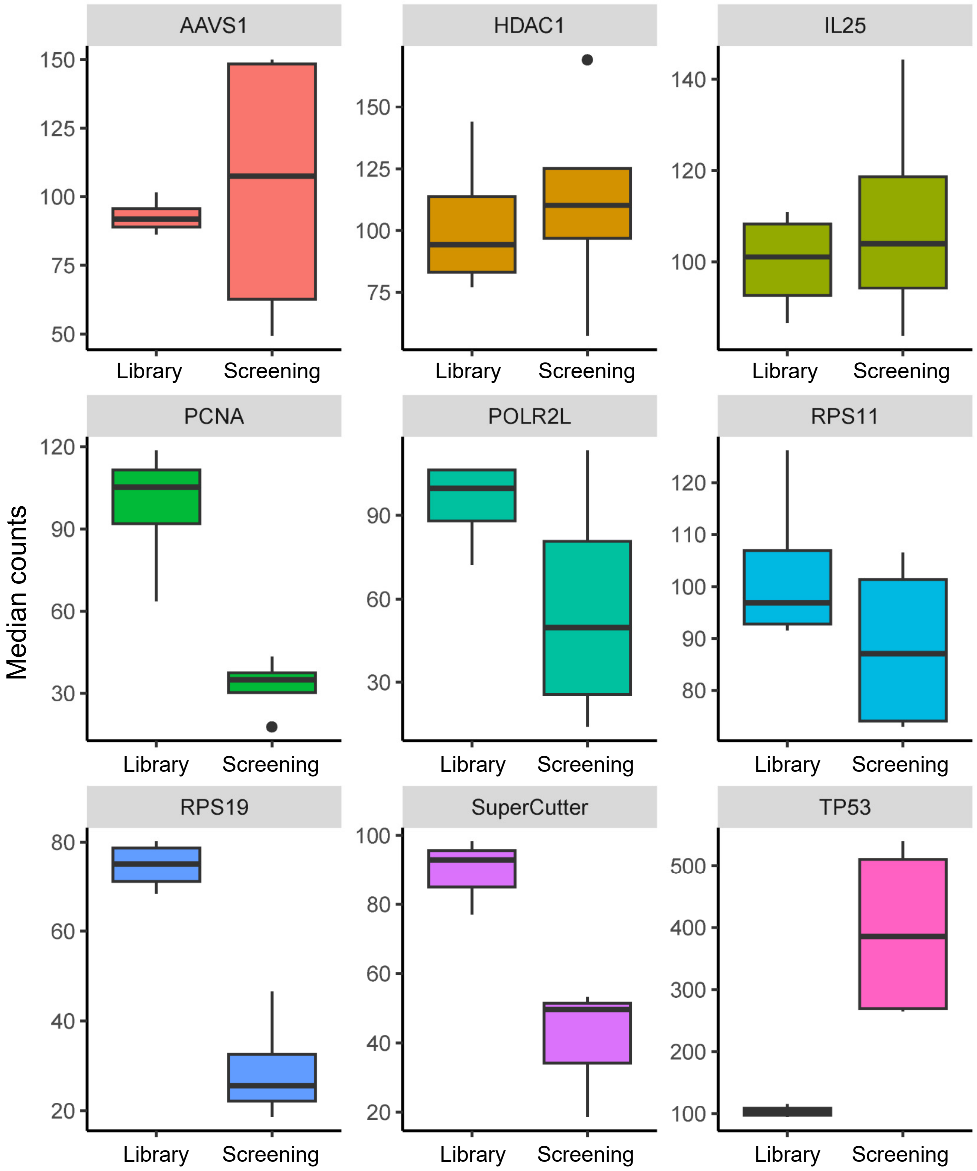

Figure S4. Effect of CRISPR-mediated targeting of control genes in human endothelial cells.

Boxplots illustrating the median counts of gRNAs directed against negative (PCNA, POLR2L, RPS11, RPS19), neutral (AAVS1, HDAC1, IL25), and positive (TP53) control genes as well as the SuperCutter (targeting highly repetitive elements in the genome) in the initial plasmid gRNA library and at the end of the CRISPR screening; n=3 biological replicates.
