# Supplementary material for: Identification of unannotated microproteins involved in endothelial cell homeostasis, dysfunction, and vascular disease
Source: Cardiovasc Res. 2026 May 19;122(9):1257–70. doi: 10.1093/cvr/cvag110 (PMC13307568; doi:10.1093/cvr/cvag110)
Supplement: cvag110_Supplementary_Data [file cvag110_supplementary_data.zip › Siragusa et al_Supplementary Methods.pdf]

SUPPLEMENTARY METHODS

Cell culture

Human umbilical vein endothelial cells were isolated and cultured as described previously,[1,2] and used up to passage 4. The use of human material in this study complies with the principles outlined in the Declaration of Helsinki (World Medical Association, 2013), and the isolation of endothelial cells was approved in written form by the ethics committee of the Goethe-University. HEK-293T and EA.hy926 cells were obtained from the American Type Culture Collection (LGC Standards, Wesel, Germany) and cultured in minimal essential medium containing 8% heat inactivated foetal bovine serum (FBS), gentamycin (25 µg/mL) non-essential amino acids (Thermo Fisher Scientific, Schwerte, Germany) and Na pyruvate (1 mM, Sigma-Aldrich, Darmstadt, Germany). All cells were negative for mycoplasma contamination. Cultured cells were kept in a humidified incubator at 37°C containing 5% CO_2.

Generation of adenoviruses

The CMV-promoter driven Rpl22-3xHA (RiboTag) construct in the pcDNA3.1(+) backbone was kindly provided by G. Stanley McKnight (University of Washington).[3] The pcDNA3.1(+)Rpl22-3xHA plasmid was digested with XbaI and NheI and ligated into a pAdShuttle-CMV plasmid, which had been digested with XbaI. The construct was verified by sequencing. Replication incompetent adenoviruses were generated using the AdEasy Adenoviral Vector System (Agilent Technologies Deutschland GmbH, Waldbronn, Germany).[4]

Adenoviral transduction and treatment of endothelial cells

Human endothelial cells (passages 2 to 4, 90% confluent) were starved of serum in MCDB131 medium (Gibco/Thermo Fisher Scientific) containing 0.1% bovine serum albumin (BSA) and transduced overnight with adenoviruses to express the RiboTag (20 MOI). The culture medium was then replaced with MCDB131 medium containing 8% heat inactivated FBS, ECGS/heparin, basic fibroblast growth factor (1 ng/mL) and epidermal growth factor (0.1 ng/mL), and the cells were allowed to recover for 10 hours before treatment with solvent (phosphate buffered saline (PBS) containing 0.1%BSA) or recombinant human IL-1β (20 ng/mL; Peprotech, Hamburg, Germany) for 16 hours. Cells were then processed for RiboTag isolation or harvested using accutase (Thermo Fisher Scientific), pelleted and snap frozen for proteomic studies.

Collection of cell culture supernatants and protein precipitation for proteomics

Human endothelial cells (passage 4, confluent) were treated with solvent (PBS containing 0.1% BSA) or recombinant human IL-1β (20 ng/mL, Peprotech, Hamburg, Germany) for 16 hours in endothelial cell growth medium 2 (ECGM2, PromoCell) containing 5% FBS and diluted 1:1 with endothelial cell basal medium 2 (EBM2, PromoCell) to reduce FBS for downstream proteomic analyses. Protein precipitation was performed as previously described[5] with modifications. Briefly, cell culture supernatants from two ∅10 cm dishes were combined and an EDTA-free protease inhibitor mix (AppliChem GmbH, Darmstadt, Germany) was added. The supernatant was then transferred to polypropylene tubes and *N*-lauroylsarcosine sodium salt and trichloroacetic acid were added to a final concentration of 0.1% and 7.5%, respectively. Samples were incubated on ice for 2 hours before being centrifuged (10,000g, 10 minutes, 4°C). The pellets were washed twice with cold tetrahydrofuran (10% of initial supernatant volume) and stored at -20°C until analysed further.

Transcoronary ablation of septal hypertrophy (TASH) patient samples

Eight patients (characteristics summarized in **Table S17**) with hypertrophic obstructive cardiomyopathy who were undergoing TASH were included in the study. The pre- and post-procedural management of these patients was as documented.[6] In brief, diagnostic assessment was performed according to the current guidelines, based on severe symptoms, asymmetrical septal hypertrophy (>15 mm), systolic movement of the anterior mitral valve leaflet, and an intraventricular pressure gradient of 30 mmHg at rest and/or 50 mmHg after Valsalva manoeuvre. TASH was performed according to standard clinical practice. All patients

provided written informed consent for their participation in the study, and the ethics board of the state of Hessen, Germany, approved the study (FF 31/2010). Venous blood samples for miP detection were collected before (baseline) and four hours after induction of myocardial infarction using TASH. Samples were processed immediately and serum was frozen at -80°C.

Human carotid artery samples

Human carotid tissue from the Munich Vascular Biobank[7] was studied and all patients had provided written and informed consent according to the Declaration of Helsinki. Tissue sampling was approved by the local ethics committee (Ethikkommission Klinikum rechts der Isar: 2799/10). Human carotid specimens were harvested during carotid endarterectomy. After removal from the intraoperative situs, tissue was immediately kept in RNA*later* Stabilization Solution (Invitrogen) at 4°C for transport and further processed in the laboratory. Samples were fixed in formalin (4% PFA) for 24 hours and if necessary, decalcification on EDTA basis (Entkalker soft SOLVAGREEN®, Carl ROTH, Karlsruhe, Germany) was performed for 2–5 days. Formalin-fixed paraffin embedded sections used for immunohistochemistry were mounted on poly-L-lysine (Merck, Darmstadt, Germany) pre-treated glass slides (SuperFrost PLUS, Epredia Europe, Basel, Suisse). All patient data are summarized in **Table S18**.

Animals

Endothelial cell-specific RiboTag mice (EC-RiboTag) were generated by crossing endothelial cell-specific Cre-driver mice (Cdh5-CreERT2)[8] with Ribo-Tag ($Rpl22^{HA/HA}$)[3] mice. The offspring were backcrossed with C57BL/6J mice (Charles River) for at least 8-10 generations. Both male and female mice were included in all studies. Cre-mediated recombination was induced in 8-9 week old mice by intraperitoneal injections of tamoxifen (50 mg/kg/day dissolved in 50 µL Miglyol; Merck, Darmstadt, Germany) for 5 consecutive days. Animals were housed under a 12 hour light-dark cycle with free access to water and a normal chow diet. One group of animals was sacrificed after deep anesthetization with 180 mg/kg ketamine and 16 mg/kg xylazine 10 days after the last tamoxifen injection. Thereafter, thorax was opened and intracardiac perfusion (through the left and right ventricles) was performed with 10 mL 100 µg/mL cycloheximide (Sigma-Aldrich) in PBS. After perfusion, vessels and organs were harvested, snap frozen in liquid nitrogen and stored at -80°C until use. Male $ApoE^{-/-}$ mice (8-10 weeks of age) were purchased from Charles River Laboratories (Sulzfeld, Germany) and partial carotid ligation was performed as described.[9] Two or seven days after ligation, animals were placed in an airtight anaesthesia box and the oxygen-isoflurane mixture was then flooded with >5% isoflurane via an isoflurane vaporiser. Animal death was confirmed by the complete cessation of breathing for at least one minute and the absence of paw reflex responses. Vessels and organs were harvested, snap frozen in liquid nitrogen and stored at -80°C until processed. All animal experiments were performed in accordance with the Directive 2010/63/EU of the European Parliament on the protection of animals used for scientific purposes and approved by the Federal Authority for Animal Research at the Regierungspräsidium Darmstadt (Hessen, Germany) under study protocols B2/1102, B2/1187 and F28/42. For experiments involving RNA-sequencing and mass spectrometry, animal sample size was decided based on previous experience with similar experiments. No animals were excluded from any of the analyses. As all animals received the same treatments and/or surgical procedure, no randomization, control of potential confounders and blinding was necessary.

AAV-PCSK9 generation and partial carotid artery ligation

An AAV serotype 8 vector for expression of the murine D377Y-PCSK9 cDNA (AAV-PCSK9) was produced using the two-plasmid-method by co-transfecting AAV/D377Y-mPCSK9 (gift from Jacob Bentzon; Addgene plasmid # 58376)[10] together with the helper plasmid pDP8[11] in HEK-293T cells using polyethylenimine (Sigma-Aldrich). AAV vectors were purified using iodixanol step gradients and titrated as described previously.[12]

Accelerated endothelial activation and dysfunction was induced as described.[13] Three to five days after the last tamoxifen injection, EC-RiboTag mice (9-10 weeks of age) were injected once with AAV-PCSK9 (10^{11} VG) via the tail vein. Thereafter, mice were fed a cholesterol rich

diet (metabolizable energy 35 kJ% fat, containing 12.5 mg/kg cholesterol; EF PAIGEN, Ssniff, Soest, Germany). One week after AAV-PCSK9 injection and initiation of the cholesterol rich diet, partial ligation of the left carotid artery was performed as described.[9] An injection of 0.1 mg/kg buprenorphine 30 minutes before surgery was used to alleviate surgical pain before, during and after awakening from anaesthesia. Metamizole (non-opioid analgesic, 1.4 mg/ml (350 mg/kg/day), total duration of 5 days) was administered via the drinking water to alleviate postoperative pain one day preoperatively and a further 3 days postoperatively. The animals were placed in an airtight anaesthesia box to induce anaesthesia, the oxygen-Isoflurane mixture was then flooded with 4% VE Isoflurane via an isoflurane vaporiser. After anaesthesia was induced, the animals were placed on a heated warming mat for the entire duration of the operation to prevent hypothermia, and the cornea of the eyes was protected from drying out with Bepanthen eye ointment. For the duration of the operation (approx. 15-20 min), the mice were sedated via a mask with approx. 1.5 -3% VE Isofluran VE as required.

RiboTag and RNA isolation

Cells were collected and lysed in buffer containing: Tris/HCl pH 7.5 (50 mM), NaCl (150 mM), $MgCl_2$ (12 mM), NP-40 (1%), DTT (1 mM), EDTA-free protease inhibitor mix (AppliChem GmbH, Darmstadt, Germany), SUPERase In RNase Inhibitor (200U/mL, Thermo Fisher Scientific), cycloheximide (100 µg/mL) and TURBO DNaseI (25U/mL, Thermo Fisher Scientific), in UltraPure DNase/RNase-free distilled water (Thermo Fisher Scientific). Lysates were passed 5 times through 26G needles and recovered by centrifugation (16,000g, 10 minutes, 4°C). Murine tissues were homogenized in lysis buffer (2-3% w/v) supplemented with murine RNase inhibitor (1000 U/mL for lung tissue, 500 U/mL for all other tissues; New England Biolabs, Frankfurt am Main, Germany) and heparin (10 mg/mL). Lysates were centrifuged (16,000g, 10 minutes, 4°C) and the supernatants passed through 70 µm pre-separation filters (Miltenyi Biotec, Bergisch Gladbach, Germany). A fraction (10%) of the total lysate volume was mixed with 9 volumes of QIAZOL and frozen at -80°C. The remaining lysates were incubated with anti-HA antibody-conjugated magnetic beads (50 µL/sample, washed and resuspended in lysis buffer, Biozol, Eching, Germany) before being incubated overnight on end-over-end rocker at 4°C. HA immunoprecipitates were washed 3 times with a freshly prepared high salt buffer containing: Tris/HCl pH 7.5 (50 mM), NaCl (300 mM), $MgCl_2$ (12 mM), NP-40 (0.5%), DTT (1 mM), SUPERase In RNase Inhibitor (200 U/mL, Thermo Fisher Scientific) and cycloheximide (100 µg/mL). For experiments using murine tissues, murine RNase inhibitor (1000 U/mL for lung tissue, 500 U/mL for all other tissues; New England Biolabs) was also included. After the final wash, HA immunoprecipitates were suspended in 700 µL QIAZOL and immediately processed for RNA purification. The total input RNA (from the total lysates) as well as the ribosome-associated RNA (from the RiboTag immunoprecipitates) were purified using the miRNeasy Micro kit (QIAGEN, Hilden, Germany), according to the manufacturer's instructions.

Next generation sequencing

smORFs. RNA and library preparation integrity were verified with a LabChip Gx Touch 24 Nucleic Acid Analyzer (Perkin Elmer, Waltham, USA). Total RNA (100 ng - 1 µg) was used as input for Truseq stranded mRNA library preparation following the low input RNA protocol (Illumina, San Diego, USA) without fragmentation (5 minutes 65°C for RT annealing). Finished libraries were size selected using the Pippin device (Sage science, Beverly, USA) for insert sizes < 300nt. Due to the low amount of RNA recovered from mouse carotid artery samples, only 10 ng of total RNA was used (SMART-Seq stranded kit, Takara Bio, Gennevilliers, France) according to the manufacturer's instructions with standard RNA fragmentation (6 minutes, +85°C) and no further size selection. Sequencing was performed on the NextSeq500 instrument (Illumina) using v2 chemistry, resulting in 8-62M reads per library (average 25M) with 1x75 nt single-end setup.

Bioinformatic analyses

QC/Trimming: Raw reads were assessed for quality, adapter content and duplication rates with FastQC 0.11.8 (Andrews S. 2010, FastQC: a quality control tool for high throughput sequence

data: http://www.bioinformatics.babraham.ac.uk/projects/fastqc). Trimmomatic version 0.39 was employed to trim reads after a quality drop below a mean of Q15 in a window of 5 nucleotides for smORF analyses or a mean of Q20 in a window of 20 nucleotides for gene analyses.[14] Only reads of at least 15 nucleotides were cleared for subsequent analyses.

Gene expression pipeline. Trimmed and filtered reads were aligned versus assembly version hg38 for human or mm10 for mouse data (Ensembl release 99 - 109) using STAR 2.6.1d or 2.7.10a to include multi-mapping reads with the parameters "--outFilterMismatchNoverLmax 0.1 --outFilterScoreMinOverLread 0.9 --outFilterMatchNminOverLread 0.9 --alignIntronMax 200000 --outFilterMultimapNmax 999".[15] The number of reads aligning to genes was counted with featureCounts 1.6.5 or 2.0.4 of the Subread package.[16] Only reads mapping at least partially inside exons were admitted and aggregated per gene. Reads overlapping multiple genes or aligning to multiple regions were excluded. Differentially expressed genes were identified using DESeq2 version 1.18.1 or 1.36.[17] Genes were classified to be significantly differentially expressed at average count > 5 with Benjamini-Hochberg corrected P value ≤ 0.05 and -0.585≤ Log2fold change ≥+0.585. The annotation was enriched with UniProt data (release 25.06.2019) based on Ensembl gene identifiers.[18]

smORF pipelines. For the transcriptome/smORF assembly, reads were mapped similarly to the general analysis described above, with the exception that reads aligning to multiple regions were not excluded. The transcriptome was reference assembled with MAPS 2.0 at a diversity score of 0.1 for separate assemblies and 0.5 for combined assemblies (larger = more sensitive).[19] ORFs were called with a start codon of ATG and 1-nt variations thereof. If this could not be found, putative ORFs were called downstream the following in-frame stop codon (alternative ORFs).[20] To ensure consistency with the experimentally observed RiboTag-sequencing reads, ORFs were not manually extended to the next downstream stop codon during the initial assembling of putative smORFs to be used in subsequent proteomic searches. However, each smORF was then assessed for the presence of a downstream in-frame stop codon (TGA, TAA, or TAG) within a maximum ORF length of 300 nucleotides. If a stop codon was identified, the original spliced sequence was retained and extended accordingly. Only smORFs between 20 and 100 amino acids were retained. These were further filtered to remove peptides found to be sub-sequences of known UniProt proteins (release 25.06.2019) at >90% amino acid identity using CD-HIT 4.8.1.[21] Two different strategies were used for assembly and quantification of smORF candidates of each dataset using RNA-seq/RiboTag: 1) separate stand-alone assemblies for each condition, 2) one combined assembly followed by differential expression analyses per contrast (RiboTag/Input, RiboTag condition 1/RiboTag condition 2, Input condition 1/Input condition 2 etc.). Putative smORFs from two independent databases are rarely identical, which complicates a direct comparison between experimental groups. The first strategy was used to establish the presence/absence of a smORF in a sample (used for cultured endothelial cells under homeostatic conditions). In the second strategy, reads covering putative smORFs were quantified per replicate using featureCounts (to ensure that only one definition of a smORF exists) and differentially expressed smORFs were identified using DESeq2. SmORFs were classified as significantly differentially expressed with Benjamini-Hochberg corrected P-value < 0.05 and Log2fold change ≤ -0.585 or ≥ +0.585. This approach also increased the total sequencing depth available to the assembler leading to higher sensitivity. This second strategy was used to generate the datasets related to the IL-1β-regulated smORFeome and the murine smORFeomes. When evaluating differential expression of smORFs overlapping protein-coding exons, reads mapping to previously annotated genes could not be distinguished from those mapping to smORFs at the same genomic location, as both give rise to the same RNA molecule or parts thereof. Altered expression of such transcripts is likely to result in altered expression of both the encoded protein and the miP. Existing tools and pipelines, e.g. riborf, orf-rater, ribotricer, ribotaper, were not used because they are specifically tailored to Ribo-Seq and not RiboTag-seq data.

smORF classification

SmORFs were classified based on their genomic location relative to annotated genes.[22] For each annotated gene, only the canonical protein-coding transcript defined as longest sum of exon sequences was used as reference. The classification includes: uORF: ORF completely within the 5' UTR of annotated protein-coding gene; uoORF: ORF that begins in the 5' UTR of annotated protein-coding gene and partially overlaps its CDS; intORF: ORF completely inside CDS of annotated protein-coding gene, but translated in a different reading frame; doORF: ORF that begins inside CDS and overlaps the 3' UTR of annotated protein-coding gene; dORF: ORF completely within the 3' UTR of annotated protein-coding gene; lncRNA-ORF: ORF that overlaps non-coding RNA gene (lncRNA, miscRNA, snRNA, miRNA, transcripts To be Experimentally Confirmed [TEC], snoRNA); intronic ORF: ORF completely within intron of annotated protein-coding gene; intergenic ORF: ORF in region without any previous gene annotation; other: ORF does not overlap an annotated UTR or begins upstream and partially overlaps with an annotated 5' UTR or is only partially intronic, i.e. start/stop within annotated CDS.

Homology analyses

Homology analysis of human/mouse datasets. Human and mouse smORF datasets were compared at the *in silico* translated peptide level with BLASTP+ 2.12.0.[23] Minimum homology was defined as E-value < 10, Word size 7, positive peptides (functionally similar: amino acids with similar functional groups conferring similar polarity, hydrophilicity or charge features) >70%, coverage >80%.

Sample preparation for miP identification by mass spectrometry

Endothelial cell pellets were suspended in lysis buffer containing 4% SDS in 10 mM Tris-HCl pH 7.6 at room temperature, vortexed and heated at 95°C for 5 minutes. After 20 cycles of gentle sonication (10%, Branson Ultrasonic, Carouge, Switzerland) at room temperature, lysates were centrifuged (16,000 g, 10 minutes, room temperature) and the protein concentration determined (Pierce 660nm Protein Assay Reagent, Thermo Fisher Scientific). Protein lysates (50 µg) were mixed with 4 x lithium dodecyl sulfate sample buffer (NuPAGE LDS Sample Buffer 4x, Thermo Fisher Scientific) and 10 mM DTT, boiled for 10 minutes at 70°C and loaded onto Bis-Tris gels (NuPAGE 4-12% Bis-Tris Gel, Thermo Fisher Scientific). The gels were cut into 6 slices per gel lane in the mass range below 30 kDa prior to in gel digestion.[24] In brief, gel samples and proteins were reduced (10 mM DTT) and then alkylated (55 mM iodoacetamide). Proteins were digested overnight with trypsin (MS approved, porcine, Serva, Heidelberg, Germany), using an enzyme to protein ratio of 1:100. Peptides were gradually eluted from the gel by increasing concentrations of acetonitrile. Samples were desalted using stop and go extraction tips[25] (stage tips: 200 µL tips filled with C18, Empore SPE disc, CDS Analytical, Oxford, USA) and analysed by liquid chromatography and tandem mass spectrometry (LC-MS/MS). Protein lysates from the same samples (50 µg) were also precipitated with four volumes of acetone (1 hour, -20°C) before being centrifuged (16,000 g, 10 minutes), followed by washing with 90% acetone. Pellets were subsequently resolved in 6 M urea and 2 M thiourea in 10 mM HEPES pH 7.4 and digested in solution with Lys-C (Lysyl Endopeptidase, Wako, Neuss, Germany) and trypsin (MS approved, porcine, Serva) at an enzyme to protein ratio of 1:100 and 1:50, respectively. Thereafter, peptides were fractionated by hydrophobicity with high pH reversed-phase columns (Pierce High pH Reversed-Phase Fractionation Kit, Thermo Fisher Scientific) and analysed by LC-MS/MS.

Murine lungs, hearts, aortae and carotid arteries (right and left carotid arteries from two mice were pooled), were ground to powder on dry ice using a mortar and pestle. Samples were then lysed in buffer containing 6 M urea and 2 M thiourea in 10mM HEPES pH 7.4 (all tissues) and either sonicated (lungs, hearts and aortae) or treated with benzonase (carotid arteries, 44 U/mL, Merck). Lysates were centrifuged (16,000 g, 10 minutes, 4°C) and the protein concentration determined. Protein lysates (50 µg) were mixed with 4 x lithium dodecyl sulfate sample buffer and 10 mM DTT and loaded onto tricine gels (Novex Tricine Protein Gels, Thermo Fisher Scientific), prior to in gel digestion. All tissue protein lysates were also processed for in solution digestion as described above. The protein lysates from mouse lung

and heart tissues were additionally processed for reversed-phase C8 cartridge-based isolation of small proteins and peptides, a method successfully applied for the detection of non-annotated miPs.[19,26,27] Briefly, protein lysates were precipitated with methanol/chloroform[28] and reconstituted in C8 lysis buffer (85% HCl, 10 mM DTT, 0.05% Triton X-100). Samples were boiled (10 minutes) and centrifuged (16,000g, 20 minutes) and the final protein concentration determined. C8 cartridges (Altmann Analytik, Munich, Germany) were preconditioned with 1 column volume (CV) methanol and 2 CV 0.25 M triethylammonium formate pH 3 (TEAF, VWR, Darmstadt, Germany) followed by sample loading. Elution was performed with 2 CV TEAF:acetonitrile 3:1 and 2 CV TEAF:acetonitrile 1:3. Eluates were pooled and dried in a vacuum concentrator. Dried peptides were dissolved in water and precipitated with methanol/chloroform. In solution digestion was performed as described above and peptides were applied to LC-MS/MS after C18 reversed phase stop and go extraction (STAGE) tips.

Precipitated protein pellets from cell culture supernatants as well as human serum samples diluted in water were reconstituted in buffer containing 6 M urea, 2 M thiourea and 10 mM HEPES pH 7.4. For cell culture pellets only benzonase (44 U/mL, Merck) was also added and samples were subsequently processed for in gel digestion (tricine gels), in solution digestion followed by high pH reversed phase fractionation as well as reversed-phase C8 cartridge-based small protein enrichment as described above and analysed by LC-MS/MS.

Liquid chromatography and tandem mass spectrometry (LC-MS/MS)

LC-MS/MS analyzes where performed using an EASY-nLC 1200 nano flow chromatography sytem interfacing with QExactive and QExactive HF mass spectrometers (all ThermoFisher Scientific, Bremen, Germany) through electrospray ionization with in house packed column emitters (20 cm, 75 µm ID, 1.9 µm C18 beads, Dr. Maisch GmbH, Ammerbuch, Germany) and using a buffer system of 0.1% formic acid (solvent A) and 80% acetonitrile/0.1% formic acid (solvent B). Relevant instrument parameters were extracted from raw data files and summarized using MARMoSET[29] and are provided in the supplemental tables of each proteomic dataset, together with the limited subset of MaxQuant parameters. Peptide/spectrum matching, protein group assembly and label free quantitation (LFQ) were performed using the MaxQuant suite of algorithms.[30–32] Full MaxQuant parametrization has been repository-deposited along with the mass spectrometric raw data. To enable the use of MaxQuant's false discovery rate (FDR) determination in the context of the large databases of *in silico* translated smORFs, a two-step process was employed. In a first step, the smORF database was concatenated with the UniProt[18] canonical and isoforms database of the organism in question, as well as MaxQuant-provided contaminant sequences. The first search was carried out without FDR control and searched without FDR control (PSM FDR: 1 and Protein FDR: 1). The resulting output was used to reduce the database to candidate smORF sequences identified by at least two unique peptides (filter applied post-MaxQuant analysis). In a second step the data was searched against the reduced database of smORFs with preliminary proteomic evidence concatenated to UniProt as above and employing a PSM FDR of 0.01 and protein FDR of 1 as reported elsewhere.[30–34] Alternatively, smORF databases of smaller size derived from differential expression analysis were directly concatenated to UniProt as above and used to search the data employing a PSM FDR of 0.01. Where differential expression analysis based on LFQ information was used (TASH samples), data where processed using the in-house developed and limma[35]-based R pipeline autonomics (https://doi.org/doi:10.18129/B9.bioc.autonomics), including quantile normalization, imputation and limma's Bayesian moderated t-testing for the identification of significant differences.

Hydropathy analysis

Grand average of hydropathy (GRAVY) scores were calculated according to Kyte and Doolittle[36] with the help of the R package *Peptides*.[37]

Prediction of miP-derived peptide binding affinities to HLA class I

To assess the potential of the identified human miPs to harbour peptides able bind to HLA class I with high affinity, we employed the latest version of netMHCpan (4.1a) as implemented

on the IEDB Analysis Resource.[38] NetMHCpan-4.1 predicts the binding of peptides to any MHC molecule with a known sequence using artificial neural networks. The method is trained on a combination of over 850,000 quantitative binding affinity and mass spectrometry eluted ligand peptides. In this study, the analysis was run on the 12 HLA class I supertypes through the DTU server (https://services.healthtech.dtu.dk/service.php?NetMHCpan-4.0). Only binders of 8-12 amino acids in length with a %rank (rank of the predicted binding score compared to a set of random natural peptides) less than 0.5 (=strong binders) were selected. MiP-derived HLA class I binders were compared to the human proteome using BLASTP+ 2.15.0 (release 01/2024): length 8 consecutive amino acids, identity 100%, coverage 100%.

High-throughput CRISPR screen

gRNA library design. CRISPR-Cas single guide RNAs (gRNAs) with high on-target and low-off-target activity were designed for each smORF and their overlapping gene transcript with the R package "Multicrispr".[39] Up to 3 non-overlapping (location and sequence) gRNAs were selected per smORF and respective overlapping gene transcript. Negative (SuperCutter, PCNA, POLR2L, RPS11, RPS19), neutral (AAVS1, HDAC1, IL25), and positive control (TP53, NF2, CDKN1B) gRNAs were also added to the library. To assess potential effects of smORFs located in intergenic regions, additional gRNAs were designed to target the intergenic region >1 kb upstream or downstream the smORF of interest. Additional controls were used to perform data quality control of the CRISPR screen e.g. effect sizes, Cohen's D, and to compare end time-point gRNA abundances with the initial library gRNA distribution (**Table S15**).

3Cs library and lentiviral plasmid generation. The smORF-specific CRISPR gRNA library was made by 3Cs, as previously reported.[40–42] Briefly, in silico-designed gRNA sequences were 5' and 3' extended by 3Cs overhangs to enable annealing to the gRNA library template plasmid and purchased from TWIST Biosciences (San Francisco, USA). The oligos were then specifically annealed to the h7SK gRNA expression cassette on pLenti_mpx_SpCas9_h7SK(PacI)_U6(I-SceI)_PGK-puro_v2 (Addgene #189632), analysed by gel electrophoresis on a 0.8% agarose gel and the successful cloning of gRNA-containing oligos into lentiviral plasmid was verified by SANGER sequencing. The quality and distribution of the plasmid gRNA library was determined by NGS on an Illumina MiSeq sequencer using a paired-end 2x74bp protocol, according to the manufacturer's instructions.

Lentivirus generation. To generate lentiviral particles, HEK293T cells (4×10^6) were seeded onto a ∅10 cm dish. After 24 hours, 2 mL Opti-MEM I (Thermo Fisher Scientific), 210 µL of GeneJuice (Sigma-Aldrich), 33 µg of the transfer vector, 27 µg of psPAX2 (Addgene: 12260) and 10 µg of pMD2.G (Addgene: 12259) were mixed, before being added dropwise to cells. After 48 hours, the supernatant was collected, aliquoted, and stored at -80 °C. To determine the titer, 50,000 RPE1 cells were seeded in a 6-well plate in 2 ml of DMEM/F12 and 8 µg/mL polybrene (Sigma-Aldrich). Cells were transduced with a ten-fold dilution series of viral supernatant (10^2 to 10^6). Two days after the transduction, puromycin (1 µg/mL, InvivoGen, Toulouse France) was added to to initiate the selection of transduced cells. Cells were maintained for 10 days before established colonies were counted and the colony number in the highest dilution was volume normalized to determine the functional lentiviral titre.

High-throughput CRISPR knockout screen. The CRISPR knockout screen was performed using the endothelial cell line EA.hy926 (n=3 biological replicates) cultured in minimal essential medium containing 8% heat inactivated FBS at a 100-fold library coverage. The total cell number for each screen was calculated by multiplying library diversity with the coverage of 100, and cells ($2,6\times10^6$) were transduced at an MOI of 0.5 (1.3 million viral units). Two days after transduction, cells were selected with puromycin (1 µg/mL) and the culture medium was replaced with medium containing puromycin. Cells were then left to grow until the end of the screen and sub-cultured at a cell number maintaining library diversity (library complexity x screen coverage) when they reached 80% confluency. Cells were harvested after 10 cell doublings, on average 14 days, after transduction for subsequent genomic DNA extraction with QIAmp DNA Blood Mini Kit (Qiagen, 51104).

NGS, data quality control and read count table generation. The amount of genomic DNA needed for coverage-based NGS library generation was determined by calculating "library complexity x experimental coverage × 6.6 pg". Genomic DNA was distributed in multiple PCR reactions (50 µL) with 25 µl of NEBNext High-Fidelity 2x PCR Master Mix, 2.5 µl of forward and reverse Illumina NGS primers (10 µM) amplifying the gRNA cassette and water. The thermal cycler parameters were set as follows: the initial denaturation at 98 °C for 5 minutes, 32 cycles of denaturation at 98 °C for 50 seconds, annealing at 68 °C for 45 seconds, and elongation at 72 °C for 1 minute, and final extension at 72 °C for 5 minutes. The PCR product was purified from a 1% agarose gel and sequenced with Illumina MiSeq according to the manufacturer's instructions. gRNA representations of the plasmid library and screen samples were determined from the FASTQ sequences obtained by the Illumina sequencing. For trimming, aligning, quality control, and read count table generation, the automated read-counting pipeline ReCo was applied.[43]

Data analysis of CRISPR knockout screen. Read count files were used as input for Model-based Analysis of Genome-wide CRISPR-Cas9 Knockout (MEGeCK).[44,45] As such, positively and negatively selected gRNAs were retrieved and associated with smORF and gene identifiers. Next, individual gRNA abundance at the end point were correlated with their initial library frequency. This step involved normalizing gRNA read counts and computing $\log_2$ fold-changes. To facilitate this comparison, MAGeCK uses a robust ranking aggregation (RRA) algorithm. This resulted in smORF and gene candidates with positive and negative $\log_2$ fold-changes and p-values that were combined pairwise for comparative visualization. A similar analysis was performed for gRNAs targeting intergenic smORFs and the corresponding control gRNAs.

Validation of CRISPR knockout screen. Plasmids and generation of lentiviruses was performed as described above. EA.hy926 cells were transduced at an MOI of 1 in the presence of 8 µg/mL polybrene. Two days after transduction, cells were selected with puromycin (3 µg/mL) for two days. The culture medium was then replaced with minimal essential medium containing 8% heat inactivated FBS and cells were allowed to grow till used. The sequences of the gRNAs used, potential off-target sites of Cas9 RNA-guided endonucleases predicted with Cas-OFFinder[46] (mismatch number=1, DNA bulge size≤1, RNA bulge size≤1) and specific primer pairs to amplify the regions around on- and off-target sites are reported in **Table S15**. Genomic DNA was isolated from wild-type EA.hy926 cells or cells 12 days after lentiviral transduction using the DNeasy Blood & Tissue Kit (Qiagen, 69504). Target loci encompassing CRISPR on- and off-target sites were amplified by PCR using Platinum Taq DNA Polymerase (Invitrogen, 10966034) and locus-specific primers (BioSpring). PCR reactions contained 50 ng of genomic DNA, 1 or 2.5 mmol/L $MgCl_2$, 200 µmol/L dNTPs and 0.4 µmol/L forward and reverse primers. Thermal cycling was performed with an initial denaturation at 94°C for 5 minutes, followed by 33 cycles of denaturation at 94 °C for 30 seconds, annealing at 60°C for 30 seconds and extension at 72°C for 30 seconds, with a final extension at 72°C for 7 minutes. PCR products were resolved on 2% agarose gels, excised at the expected amplicon size and purified using the GenCatch Advanced Gel Extraction Kit (Poch Life Sciences, 22-60250). Purified amplicons were processed for Illumina short-read next-generation sequencing (Amplicon-EZ, Genewiz, Azenta Life Sciences). The percentage of reads containing indels and indel-induced frameshifts among all target reads was determined by aligning sequencing reads to the reference human genome at the corresponding loci. Amplicons from wild-type EA.hy926 cells exhibited an apparent indel rate between 8 and 29% due to sequencing and alignment artifacts at amplicon termini. Therefore, editing frequencies at on-target and predicted off-target loci were background-corrected by subtracting the indel or frameshift rate observed in wild-type EA.hy926 cells on a per-amplicon and per-sequencing basis. Raw values are provided in **Table S15**.

Cell proliferation. Six days after transduction, cells were seeded into 96 well plates at a density of 15,000 cells/well. Cell confluency was determined for up to 3 days using an IncuCyte S3 live-cell analysis system (Sartorius, Germany). Alternatively, cells were incubated with with 10 µM 5-Ethynyl-2´-deoxyuridine (EdU) for 2 hours at 37°C. Cells were then washed with PBS, fixed with 4% Rotifix (15 minutes, room temperature), washed with 3% BSA/PBS and permeabilized with 0.5% Triton X-100 in PBS for 20 minutes. EdU-positive cells were detected using the Click-iT EdU Imaging Kit (Cat. No.: C10337, Thermo Fisher Scientific) according to the manufacturer's instructions. The number of total and EdU-positive cells was quantified using the IncuCyte S3 live-cell analysis system (Sartorius, Germany).

Cell death. Six days after transduction, cells were seeded at a density of 15,000 cells/well in 96-well plates. After four hours, the medium was replaced with culture medium supplemented with propidium iodide (PI, 10 µg/mL; Sigma-Aldrich, P4170). PI-positive cells were imaged and quantified for up to five days using the Incucyte S3 live-cell analysis system (Sartorius, Germany). The number of dead cells/well was subtracted from the number of dead cells at time 0.

Generation of a custom antibody against miP-PSTPIP2

The rabbit polyclonal antibody against miP-PSTPIP2 was generated by Eurogentec (Belgium). Rabbits were immunised with the synthetic peptide CQPGEPEATRKAF corresponding to amino acids 9-21 of miP-PSTPIP2 coupled with the carrier protein Keyhole Limpet Hemocyanin (KLH). A BLASTP search of the antigen peptide sequence against the human UniProt reference database retrieved hits with only partial query coverage and/or low percent identity indicating that it was unlikely that antibodies generated recognised antigens present in other protein sequences. Antigen-specific antibodies were purified by affinity purification from rabbit sera and the affinity profile was tested by indirect ELISA against the antigen peptide (**Figure S2**). Briefly, 5-15 µg/well of miP-PSTPIP2 peptide antigen or 100 ng/well of the KLH carrier were coated into the wells of an ELISA plate for 16 hours at 4°C. After blocking with 1 mg/mL BSA for 2 hours at 25°C, increasing dilutions of either the serum from a rabbit immunized with the miP-PSTPIP2 peptide before affinity purification, the purified antibody or the flow through were incubated for 2 hours at 25°C. The colorimetric development was performed using a secondary HRP-conjugated antibody, and o-phenylenediamine as chromogenic substrate. The optical density of the chromogenic substrate is measured at 492 nm. The specificity of the antibody against miP-PSTPIP2 was also experimentally tested by immunofluorescence in human endothelial cells after siRNA-mediated knockdown of PSTPIP2 or CRISPR/Cas9 mediated knockout of the smORF-PSTPIP2 locus (**Figure 5B-E**).

PSTPIP2 knockdown

Human endothelial cells (passage 3) were seeded in 12 well-plates at a confluency of 70%. Twenty-four hours after seeding, transfection was performed using a total concentration of 10 pM siRNA targeting PSTPIP2, consisting of 5 pM per siRNA (SI02665649 and SI02665656, Qiagen, Germany) or a non-targeting control siRNAs (siCTL, 5'-UUCUCCGAACGUGGCACGA-3' and 5'-UCGUGCCACGUUCGGAGAA-3', Eurogentec). The siRNA:Lipofectamine™ RNAiMAX Transfection Reagent (Cat. Nr.: 13778150, Invitrogen) ratio was 1:0.3 according to the manufacturer's instructions. After 5 hours, the medium was replaced with fresh ECGM2 and transfected cells were seeded for assays after 24 hours.

CRISPR-mediated knockout of smORF-PSTPIP2

Plasmid and lentivirus generation was performed as described in "High-throughput CRISPR screen". The sequence of the gRNA against the smORF-PSTPIP2 was: AACTTCAAAGACCGCAGTAG. Human endothelial cells (passage 1) at a confluency of 70% were transduced at an MOI of 0.5 in the presence of 8 µg/mL polybrene. One day after transduction, the culture medium was replaced with ECGM2 containing 5% FBS. On day 2 and 5 after transduction, the culture medium was replaced with ECGM2 containing 5% FBS and puromycin (5 µg/mL). On day 7 after transduction and every other day thereafter, the culture

media was replaced with ECGM2 containing 5% FBS. Cells were used in further experiments after at least 11 days after transduction.

Immunoblotting

Protein samples were separated by SDS–PAGE and then transferred to 0.45 mm nitrocellulose membranes (GE Healthcare, Freiburg, Germany). Membranes were incubated overnight with primary antibodies against PSTPIP2 (1:3000, Merck, HPA040944), miP-PSTPIP2 (1:500, Eurogentec), Hsp90 (1:1000, BD Biosciences, 610419), β-actin (1:1000, Sigma-Aldrich, A1978), β-tubulin (1:1000, Cell Signaling Technology, 86298) or control rabbit IgG (1:500, Merck, NI01). Thereafter, membranes were incubated with species-specific secondary antibodies anti-IgG conjugated with horseradish peroxidase. Proteins were visualized by enhanced chemiluminescence using a commercially available kit (GE Healthcare).

Immunofluorescence

Cells grown on 8 well chamber slides (Ibidi, Martinsried, Germany) were washed with PBS containing 0.8 mM $CaCl_2$ and 1.4 mM $MgCl_2$ and fixed in 4% paraformaldehyde (Carl Roth). Cells were incubated in blocking/permeabilization buffer containing 5% horse serum/0.1% Triton X-100 in PBS for 30 minutes at room temperature. A custom antibody against miP-PSTPIP2 (5 µg/mL, Eurogentec) or control normal rabbit IgG (5µg/mL, Millipore, NI01) in PBS containing 0.5% horse serum/0.01% Triton X-100 were incubated for 2 hours at room temperature. Cells were then incubated with donkey anti-rabbit Alexa Fluor 488 (1:300, Thermo Fisher Scientific) in PBS for 1 hour at room temperature. Nuclei were stained using DAPI (Thermo Fisher Scientific) before mounting with mounting medium (43% Glycerol, 100 mM DTT). Images were taken using a confocal microscope (SP8; Leica, Wetzlar, Germany) and LAS AF lite software (Leica).

Carotid arteries were embedded together with the aortic arch in Tissue-Tek optimum cutting temperature medium (VWR, Darmstadt, Germany), frozen on dry ice, and stored at -80°C. Frozen sections (5 to 10 µm) were fixed in 4% paraformaldehyde (Carl Roth) for 10 minutes at room temperature before staining. Samples were then incubated in blocking buffer containing 5% donkey serum, 1% BSA (Sigma-Aldrich) and 0.1% Triton X-100 in PBS) for 1 hour at room temperature. To identify miP-PSTPIP2, samples were incubated with the custom antibody (5 µg/mL), as well as with anti-CD31 (0.08 µg/mL, BD Biosciences, Cat. Nr. 550274), control normal rabbit IgG (5µg/mL, Millipore, NI01) or control rat IgG (0.08 µg/mL, Santa Cruz, sc-2026) overnight at 4°C. This was followed by appropriate secondary antibodies i.e., donkey anti-rabbit Alexa Fluor 555 or donkey anti-rat Alexa Fluor 647 (1:300, Thermo Fisher Scientific) in phosphate-buffered saline for 1 hour (room temperature). Nuclei were stained using DAPI (Thermo Fisher Scientific) before mounting with Fluoromount-G mounting medium (Thermo Fisher Scientific). Z-stack images were taken using a confocal microscope (SP8; Leica, Wetzlar, Germany) and LAS AF lite software (Leica). Image processing and analysis were done with ImageJ. The CD31+ endothelial cells were used to create a selection for quantification of the mean gray values of the endothelial miP-PSTPIP2 signal in the image of the miP-PSTPIP2 signal. Mean gray values were finally expressed as fold change of $ApoE^{-/-}$ RCA.

Human carotid sections were obtained from the Vascular Biobank of the Technical University Munich as formalin-fixed, paraffin-embedded sections. These samples were incubated at 60°C for 10 minutes followed by deparaffinization in 100% xylene. Slices were transferred to a series of descending ethanol concentrations and finally to distilled water for 5 minutes each (100%, 96%, 90%, 80%, 70% and 50%). The samples were soaked in pre-heated antigen retrieval solution (10 mM sodium citrate pH 6) at 90–100 °C for 20 minutes. After washing with distilled water, samples were blocked with PBS containing 5% horse serum, 3% BSA and 0.1% Triton X-100 (1 hour, room temperature). Sections were then incubated with anti-miP-PSTPIP2 (5 µg/mL, Eurogentec), and Ulex (biotinylated Ulex Europaeus Agglutinin I, 1:200, Vector Laboratories) in PBS containing 0.5% horse serum, 0.3% BSA and 0.01% Triton X-100 (overnight, 4°C). After washing with PBS, the samples were incubated with donkey anti-rabbit Alexa Flour 555 secondary antibody (1:300, Thermo Fisher Scientific), Alexa Flour 647-

conjugated streptavidin (1:300, Thermo Fisher Scientific) and DAPI (1:300) diluted in PBS (2 hours, room temperature). Finally, samples were washed four times with PBS before mounting with Fluoromount-G (Thermo Fisher Scientific). Images were taken using a confocal microscope (LSM-780, Zeiss, Jena, Germany) and ZEN software (Zeiss).

REFERENCES

1. Fleming I, Fisslthaler B, Dixit M, Busse R. Role of PECAM-1 in the shear-stress-induced activation of Akt and the endothelial nitric oxide synthase (eNOS) in endothelial cells. *J Cell Sci* 2005;**118**:4103–4111.
2. Busse R, Lamontagne D. Endothelium-derived bradykinin is responsible for the increase in calcium produced by angiotensin-converting enzyme inhibitors in human endothelial cells. *Naunyn-Schmiedeberg's Arch Pharmacol* 1991;**344**:126–129.
3. Sanz E, Yang L, Su T, Morris DR, McKnight GS, Amieux PS. Cell-type-specific isolation of ribosome-associated mRNA from complex tissues. *Proc Natl Acad Sci USA* 2009;**106**:13939–13944.
4. He TC, Zhou S, da Costa LT, Yu J, Kinzler KW, Vogelstein B. A simplified system for generating recombinant adenoviruses. *Proc Natl Acad Sci USA* 1998;**95**:2509–2514.
5. Chevallet M, Diemer H, van Dorssealer A, Villiers C, Rabilloud T. Toward a better analysis of secreted proteins: the example of the myeloid cells secretome. *Proteomics* 2007;**7**:1757–1770.
6. Liebetrau C, Möllmann H, Nef H, Szardien S, Rixe J, Troidl C, Willmer M, Hoffmann J, Weber M, Rolf A, Hamm C. Release kinetics of cardiac biomarkers in patients undergoing transcoronary ablation of septal hypertrophy. *Clin Chem* 2012;**58**:1049–1054.
7. Pelisek J, Hegenloh R, Bauer S, Metschl S, Pauli J, Glukha N, Busch A, Reutersberg B, Kallmayer M, Trenner M, Wendorff H, Tsantilas P, Schmid S, Knappich C, Schaeffer C, Stadlbauer T, Biro G, Wertern U, Meisner F, Stoklasa K, Menges A-L, Radu O, Dallmann-Sieber S, Karlas A, Knipfer E, Reeps C, Zimmermann A, Maegdefessel L, Eckstein H-H. Biobanking: Objectives, Requirements, and Future Challenges-Experiences from the Munich Vascular Biobank. *J Clin Med* 2019;**8**.
8. Pitulescu ME, Schmidt I, Benedito R, Adams RH. Inducible gene targeting in the neonatal vasculature and analysis of retinal angiogenesis in mice. *Nat Protoc* 2010;**5**:1518–1534.
9. Siragusa M, Thöle J, Bibli S-I, Luck B, Loot AE, Silva K de, Wittig I, Heidler J, Stingl H, Randriamboavonjy V, Kohlstedt K, Brüne B, Weigert A, Fisslthaler B, Fleming I. Nitric oxide maintains endothelial redox homeostasis through PKM2 inhibition. *EMBO J* 2019;**38**:e100938.
10. Bjørklund MM, Hollensen AK, Hagensen MK, Dagnaes-Hansen F, Christoffersen C, Mikkelsen JG, Bentzon JF. Induction of atherosclerosis in mice and hamsters without germline genetic engineering. *Circ Res* 2014;**114**:1684–1689.
11. Sonntag F, Köther K, Schmidt K, Weghofer M, Raupp C, Nieto K, Kuck A, Gerlach B, Böttcher B, Müller OJ, Lux K, Hörer M, Kleinschmidt JA. The assembly-activating protein promotes capsid assembly of different adeno-associated virus serotypes. *J Virol* 2011;**85**:12686–12697.
12. Jungmann A, Leuchs B, Rommelaere J, Katus HA, Müller OJ. Protocol for efficient generation and characterization of adeno-associated viral vectors. *Hum Gene Ther Methods* 2017;**28**:235–246.

13. Kumar S, Kang D-W, Rezvan A, Jo H. Accelerated atherosclerosis development in C57Bl6 mice by overexpressing AAV-mediated PCSK9 and partial carotid ligation. *Lab Invest* 2017;**97**:935–945.
14. Bolger AM, Lohse M, Usadel B. Trimmomatic: a flexible trimmer for Illumina sequence data. *Bioinformatics* 2014;**30**:2114–2120.
15. Dobin A, Davis CA, Schlesinger F, Drenkow J, Zaleski C, Jha S, Batut P, Chaisson M, Gingeras TR. STAR: ultrafast universal RNA-seq aligner. *Bioinformatics* 2013;**29**:15–21.
16. Liao Y, Smyth GK, Shi W. featureCounts: an efficient general purpose program for assigning sequence reads to genomic features. *Bioinformatics* 2014;**30**:923–930.
17. Love MI, Huber W, Anders S. Moderated estimation of fold change and dispersion for RNA-seq data with DESeq2. *Genome Biol* 2014;**15**:550.
18. UniProt Consortium. Activities at the Universal Protein Resource (UniProt). *Nucleic Acids Res* 2014;**42**:D191-8.
19. Ma J, Saghatelian A, Shokhirev MN. The influence of transcript assembly on the proteogenomics discovery of microproteins. *PLoS One* 2018;**13**:e0194518.
20. Cao X, Slavoff SA. Non-AUG start codons: Expanding and regulating the small and alternative ORFeome. *Exp Cell Res* 2020;**391**:111973.
21. Li W, Godzik A. Cd-hit: a fast program for clustering and comparing large sets of protein or nucleotide sequences. *Bioinformatics* 2006;**22**:1658–1659.
22. Mudge JM, Ruiz-Orera J, Prensner JR, Brunet MA, Calvet F, Jungreis I, Gonzalez JM, Magrane M, Martinez TF, Schulz JF, Yang YT, Albà MM, Aspden JL, Baranov PV, Bazzini AA, Bruford E, Martin MJ, Calviello L, Carvunis A-R, Chen J, Couso JP, Deutsch EW, Flicek P, Frankish A, Gerstein M, Hubner N, Ingolia NT, Kellis M, Menschaert G, Moritz RL, Ohler U, Roucou X, Saghatelian A, Weissman JS, van Heesch S. Standardized annotation of translated open reading frames. *Nat Biotechnol* 2022;**40**:994–999.
23. Altschul SF, Gish W, Miller W, Myers EW, Lipman DJ. Basic local alignment search tool. *J Mol Biol* 1990;**215**:403–410.
24. Shevchenko A, Tomas H, Havlis J, Olsen JV, Mann M. In-gel digestion for mass spectrometric characterization of proteins and proteomes. *Nat Protoc* 2006;**1**:2856–2860.
25. Rappsilber J, Ishihama Y, Mann M. Stop and go extraction tips for matrix-assisted laser desorption/ionization, nanoelectrospray, and LC/MS sample pretreatment in proteomics. *Analyt Chem* 2003;**75**:663–670.
26. D'Lima NG, Ma J, Winkler L, Chu Q, Loh KH, Corpuz EO, Budnik BA, Lykke-Andersen J, Saghatelian A, Slavoff SA. A human microprotein that interacts with the mRNA decapping complex. *Nat Chem Biol* 2017;**13**:174–180.
27. Khitun A, Slavoff SA. Proteomic detection and validation of translated small open reading frames. *Curr Protoc Chem Biol* 2019;**11**:e77.
28. Wessel D, Flügge UI. A method for the quantitative recovery of protein in dilute solution in the presence of detergents and lipids. *Anal Biochem* 1984;**138**:141–143.
29. Kiweler M, Looso M, Graumann J. MARMoSET - Extracting Publication-ready Mass Spectrometry Metadata from RAW Files. *Mol Cell Proteomics* 2019;**18**:1700–1702.
30. Cox J, Hein MY, Luber CA, Paron I, Nagaraj N, Mann M. Accurate proteome-wide label-free quantification by delayed normalization and maximal peptide ratio extraction, termed MaxLFQ. *Mol Cell Proteomics* 2014;**13**:2513–2526.

31. Cox J, Mann M. MaxQuant enables high peptide identification rates, individualized p.p.b.-range mass accuracies and proteome-wide protein quantification. *Nat Biotechnol* 2008;**26**:1367–1372.
32. Cox J, Neuhauser N, Michalski A, Scheltema RA, Olsen JV, Mann M. Andromeda: a peptide search engine integrated into the MaxQuant environment. *J Proteome Res* 2011;**10**:1794–1805.
33. Ouspenskaia T, Law T, Clauser KR, Klaeger S, Sarkizova S, Aguet F, Li B, Christian E, Knisbacher BA, Le PM, Hartigan CR, Keshishian H, Apffel A, Oliveira G, Zhang W, Chen S, Chow YT, Ji Z, Jungreis I, Shukla SA, Justesen S, Bachireddy P, Kellis M, Getz G, Hacohen N, Keskin DB, Carr SA, Wu CJ, Regev A. Unannotated proteins expand the MHC-I-restricted immunopeptidome in cancer. *Nat Biotechnol* 2022;**40**:209–217.
34. Chen J, Brunner A-D, Cogan JZ, Nuñez JK, Fields AP, Adamson B, Itzhak DN, Li JY, Mann M, Leonetti MD, Weissman JS. Pervasive functional translation of noncanonical human open reading frames. *Science* 2020;**367**:1140–1146.
35. Ritchie ME, Phipson B, Di Wu, Hu Y, Law CW, Shi W, Smyth GK. limma powers differential expression analyses for RNA-sequencing and microarray studies. *Nucleic Acids Res* 2015;**43**:e47.
36. Kyte J, Doolittle RF. A simple method for displaying the hydropathic character of a protein. *J Mol Biol* 1982;**157**:105–132.
37. Osorio D, Rondón-Villarreal P, Torres R. Peptides: A Package for Data Mining of Antimicrobial Peptides. *The R Journal* 2015;**7**:4.
38. Reynisson B, Alvarez B, Paul S, Peters B, Nielsen M. NetMHCpan-4.1 and NetMHCIIpan-4.0: improved predictions of MHC antigen presentation by concurrent motif deconvolution and integration of MS MHC eluted ligand data. *Nucleic Acids Res* 2020;**48**:W449-W454.
39. Bhagwat AM, Graumann J, Wiegandt R, Bentsen M, Welker J, Kuenne C, Preussner J, Braun T, Looso M. multicrispr: gRNA design for prime editing and parallel targeting of thousands of targets. *Life Sci Alliance* 2020;**3**.
40. Wegner M, Diehl V, Bittl V, Bruyn R de, Wiechmann S, Matthess Y, Hebel M, Hayes MGB, Schaubeck S, Benner C, Heinz S, Bremm A, Dikic I, Ernst A, Kaulich M. Circular synthesized CRISPR/Cas gRNAs for functional interrogations in the coding and noncoding genome. *Elife* 2019;**8**.
41. Wegner M, Husnjak K, Kaulich M. Unbiased and tailored CRISPR/Cas gRNA libraries by synthesizing covalently-closed-circular (3Cs) DNA. *Bio Protoc* 2020;**10**.
42. Diehl V, Wegner M, Grumati P, Husnjak K, Schaubeck S, Gubas A, Shah VJ, Polat IH, Langschied F, Prieto-Garcia C, Müller K, Kalousi A, Ebersberger I, Brandts CH, Dikic I, Kaulich M. Minimized combinatorial CRISPR screens identify genetic interactions in autophagy. *Nucleic Acids Res* 2021;**49**:5684–5704.
43. Wegner M, Kaulich M. ReCo: automated NGS read-counting of single and combinatorial CRISPR gRNAs. *Bioinformatics* 2023;**39**.
44. Li W, Köster J, Xu H, Chen C-H, Xiao T, Liu JS, Brown M, Liu XS. Quality control, modeling, and visualization of CRISPR screens with MAGeCK-VISPR. *Genome Biol* 2015;**16**.
45. Li W, Xu H, Xiao T, Le Cong, Love MI, Zhang F, Irizarry RA, Liu JS, Brown M, Liu XS. MAGeCK enables robust identification of essential genes from genome-scale CRISPR/Cas9 knockout screens. *Genome Biol* 2014;**15**.
46. Bae S, Park J, Kim J-S. Cas-OFFinder: a fast and versatile algorithm that searches for potential off-target sites of Cas9 RNA-guided endonucleases. *Bioinformatics* 2014;**30**:1473–1475.
